# Functional and connectivity correlates associated with Parkinson’s disease psychosis: a systematic review

**DOI:** 10.1093/braincomms/fcae358

**Published:** 2024-11-06

**Authors:** Sara Pisani, Brandon Gunasekera, Yining Lu, Miriam Vignando, Dominic Ffytche, Dag Aarsland, K R Chaudhuri, Clive Ballard, Jee-Young Lee, Yu Kyeong Kim, Latha Velayudhan, Sagnik Bhattacharyya

**Affiliations:** Division of Academic Psychiatry, Department of Psychosis Studies, Institute of Psychiatry, Psychology and Neuroscience, King’s College London, London SE5 8AF, UK; Division of Academic Psychiatry, Department of Psychosis Studies, Institute of Psychiatry, Psychology and Neuroscience, King’s College London, London SE5 8AF, UK; Department of Psychological Medicine, Institute of Psychiatry, Psychology and Neuroscience, King’s College London, London SE5 8AF, UK; Centre for Neuroimaging Science, Institute of Psychiatry, Psychology and Neuroscience, King’s College London, London SE5 8AF, UK; Division of Academic Psychiatry, Department of Psychological Medicine, Centre for Healthy Brain Ageing, Institute of Psychiatry, Psychology and Neuroscience, King’s College London, London SE5 8AF, UK; Division of Academic Psychiatry, Department of Psychological Medicine, Centre for Healthy Brain Ageing, Institute of Psychiatry, Psychology and Neuroscience, King’s College London, London SE5 8AF, UK; Centre for Age-Related Medicine (SESAM), Stavanger University Hospital, Stavanger 4011, Norway; Department of Neurosciences, Institute of Psychiatry, Psychology and Neuroscience, and Parkinson’s Foundation Centre of Excellence, King’s College Hospital, London SE5 9RS, UK; Faculty of Health and Life Sciences, University of Exeter, Exeter EX1 2LU, UK; Department of Neurology, Seoul National University-Seoul Metropolitan Government, Boramae Medical Center, Seoul 07061, Republic of Korea; Department of Nuclear Medicine, Seoul National University-Seoul Metropolitan Government, Boramae Medical Center, Seoul 07061, Republic of Korea; Division of Academic Psychiatry, Department of Psychological Medicine, Centre for Healthy Brain Ageing, Institute of Psychiatry, Psychology and Neuroscience, King’s College London, London SE5 8AF, UK; Division of Academic Psychiatry, Department of Psychosis Studies, Institute of Psychiatry, Psychology and Neuroscience, King’s College London, London SE5 8AF, UK

**Keywords:** Parkinson’s disease, psychosis, neural correlates, systematic review, neuroimaging

## Abstract

Neural underpinnings of Parkinson’s disease psychosis remain unclear to this day with relatively few studies and reviews available. Using a systematic review approach, here, we aimed to qualitatively synthesize evidence from studies investigating Parkinson’s psychosis-specific alterations in brain structure, function or chemistry using different neuroimaging modalities. PubMed, Web of Science and Embase databases were searched for functional MRI (task-based and resting state), diffusion tensor imaging, PET and single-photon emission computed tomography studies comparing Parkinson’s disease psychosis patients with Parkinson’s patients without psychosis. We report findings from 29 studies (514 Parkinson’s psychosis patients, mean age ± SD = 67.92 ± 4.37 years; 51.36% males; 853 Parkinson’s patients, mean age ± SD = 66.75 ± 4.19 years; 55.81% males). Qualitative synthesis revealed widespread patterns of altered brain function across task-based and resting-state functional MRI studies in Parkinson’s psychosis patients compared with Parkinson’s patients without psychosis. Similarly, white matter abnormalities were reported in parietal, temporal and occipital regions. Hypo-metabolism and reduced dopamine transporter binding were also reported whole brain and in sub-cortical areas. This suggests extensive alterations affecting regions involved in high-order visual processing and attentional networks.

## Teaser

This systematic review aimed at summarizing studies on functional and connectivity correlates of Parkinson’s disease psychosis. Pisani *et al*. reviewed 29 studies, revealing altered functional substrates encompassing mostly attentional networks across occipital, parietal and temporal areas. This underscores the need for comprehensive and multiple neuroimaging approaches in future research.

## Graphical Abstract

**Graphical Abstract:**
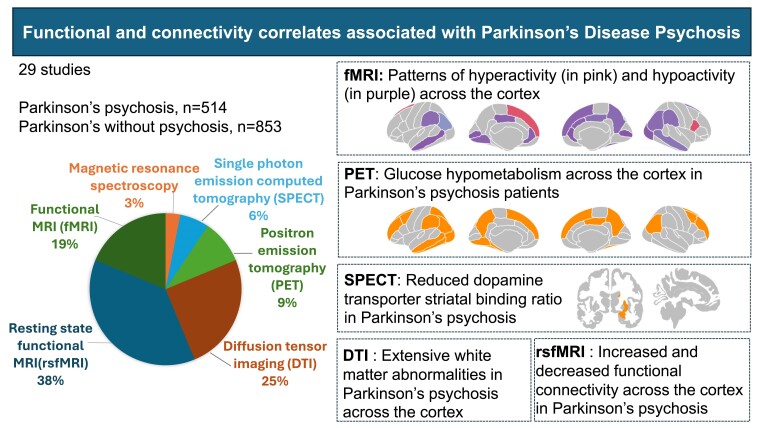

## Introduction

Parkinson’s disease is one of the most common neurodegenerative conditions worldwide, with an estimate of 9.3 million people suffering from it by 2030.^1^ Although Parkinson’s disease is mostly recognized as a movement disorder characterized by bradykinesia, tremors, rigidity and postural instability, there are in addition several non-motor symptoms (e.g. depression, constipation and urine problems, sleep issues and many others) that negatively impact patients’ quality of life.^2-5^ Specifically, Parkinson’s disease patients may experience symptoms such as hallucinations and delusions, which are collectively referred to as Parkinson’s disease psychosis (PDP), which are distressing, debilitating and associated with increased burden for carers and risk of hospitalization.^6-8^ Severity and duration of Parkinson’s disease, dopaminergic medications, sleep disorders, cognitive decline, widespread Lewy body pathology and late Parkinson’s disease onset are recognized as risk factors for developing psychotic symptoms.^8-12^

Evidence from individual studies employing different neuroimaging modalities has started to uncover the mechanisms underlying the emergence of psychotic symptoms in Parkinson’s disease. Results from structural MRI studies have reported smaller whole-brain cortical volume^13-15^ in PDP compared with Parkinson’s disease patients without psychosis (PDnP), especially in regions encompassing the ventral and dorsal visual pathways, in the parietal, temporal and occipital cortex,^16^ as well as the hippocampus.^17-19^ Complementing this, task-based and resting-state functional MRI (fMRI) studies have reported altered temporo-parietal–occipital activation and connectivity, and research using PET and diffusion tensor imaging (DTI) has also observed abnormal metabolism and structural connectivity in large-scale brain networks, respectively.^20-26^ Most reviews on the topic have focused on structural correlates involved in PDP,^27^ and few have examined its neuro-functional substrates.^28^ Integrating evidence from different neuroimaging modalities may help unravel altered mechanisms in PDP, though this may be a challenging undertaking due to different techniques and methodologies. As a number of recent reviews on the structural correlates of PDP exist^17,19^ including one from our group,^16^ here, we primarily focused on summarizing evidence from studies using other neuroimaging modalities. In particular, we have endeavoured to qualitatively synthesize evidence from separate studies employing a range of neuroimaging modalities (other than structural MRI) such as task-based and resting-state fMRI (rsfMRI), PET and DTI. The aim of this review was to summarize the main patterns of abnormal activation, connectivity and metabolism in PDP compared with PDnP patients, to provide a more comprehensive account of existing evidence than may be inferred from individual studies regarding the neural correlates of PDP that may complement evidence synthesized from structural MRI studies.

## Materials and methods

### Search strategy and eligibility criteria

We followed the Preferred Reporting Items for Systematic Reviews and Meta-Analyses guidelines^29^ (registration number: CRD42020221904), and a detailed description of the search strategy is outlined in the Supplementary material (‘Search strategy’). We systematically searched PubMed, Web of Science, Embase and the Neurosynth database on 25 January 2023, and we included peer-reviewed published studies if (i) they examined brain alterations associated with psychotic symptoms (i.e. hallucinations and/or delusions) using neuroimaging techniques [i.e. task-based fMRI, rsfMRI, PET, single-photon emission computed tomography (SPECT), DTI and magnetic resonance spectroscopy (MRS)], (ii) they included results for PDP and PDnP patients, (iii) the patients included in the studies had psychotic symptoms after Parkinson’s disease diagnosis, and (iv) the study used a case–control design. Grey literature, reviews, books and chapters, editorial and letters, conference abstracts and non-English studies were excluded from the final pool. Systematic reviews and meta-analyses were used as additional sources to search for further studies.

### Data extraction and synthesis

Data extraction involved study details (e.g. authors’ names, year of publications), study design, neuroimaging modality as well as scanner characteristics (e.g. strength, manufacturer), sample size, sample characteristics (e.g. age, gender, education levels), Parkinson’s disease onset age, disease duration, Parkinson’s disease symptoms, clinical measures of psychosis, cognitive measures, Parkinson’s disease medications [expressed in Levodopa equivalent daily dose (LEDD)], list of any other concurrent medications and any other clinical outcome measures (e.g. depression). Data extraction was conducted independently by two researchers (S.P. and Y.L.) with discrepancies addressed through consensus or discussion with senior researchers. The purpose of this review was to specifically examine evidence from neuroimaging modalities other than the structural MRI approach. Therefore, we focused on task-based fMRI, rsfMRI, PET, SPECT, MRS and DTI studies. Due to the lack of comparable methodological approaches^30^ [e.g. tract-based spatial statistics (TBSS) versus fractional anisotropy (FA) for DTI studies; whole-brain analysis versus region of interest (ROI) analysis] that would allow meaningful synthesis of data, we synthesized the results from these studies in a qualitative manner. Results were specifically reviewed to compare PDP and PDnP patients. Studies that included multiple neuroimaging modalities were considered individual studies for each modality they used. To facilitate the qualitative synthesis of evidence from the included studies, we represented visually the patterns of abnormalities in cortical and sub-cortical areas in PDP compared with PDnP patients using *ggseg*^31^ on R (version 4.0.3)^32^ and formatted using the Desikan–Killiany brain atlas.^33^

### Quality rating assessment

Study quality was assessed with the Newcastle–Ottawa rating scale for case–control studies,^34^ which assesses three methodological domains using a star rating system: ‘Selection’ (including items on case and control definition, and representativeness of cases and selection of controls, with a study receiving a rating of one star on each item, if adequate), ‘Comparability’ (i.e. whether cases and controls were comparable, with a maximum rating of two stars) and ‘Exposure’ (items on ascertainment of exposure or condition, whether the ascertainment method was comparable for cases and controls and rate of non-response, with a study receiving a rating of one star on each item if adequate). In this review, PDP patients were compared with PDnP patients; thus, the latter acted as a control or comparison group.

## Results

### Study characteristics

**Figure 1:**
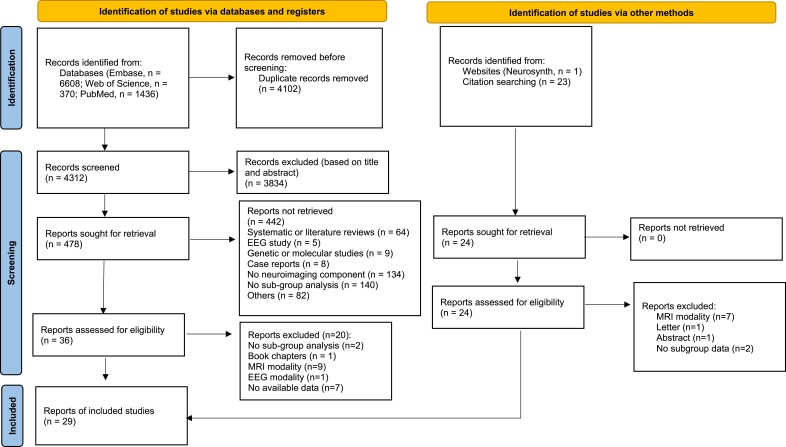
**Preferred Reporting Items for Systematic Reviews and Meta-Analyses flowchart with the study selection procedures.** Flow diagram representing study selection and inclusion process following the Preferred Reporting Items for Systematic Reviews and Meta-Analyses guidelines.

Our systematic search identified 16 studies,^13,21,23,26,35-46^ with an additional 12 studies^22,24,47-56^ identified from systematic reviews and 1^57^ from the Neurosynth database. Thus, we included a total of 29 published articles (Fig. 1) reporting on a total of *n* = 1367 patients, of which 514 had psychotic symptoms (mean age ± SD = 67.92 ± 4.37 years; 51.36% males). A total of 853 PDnP patients did not have such symptoms (mean age ± SD = 66.75 ± 4.19 years; 55.81% males) and acted as the ‘control group’. Of the 29 studies, two studies employed multi-modal neuroimaging approaches,^40,48^ and each modality was considered as an independent study. Full study characteristics are reported in Supplementary Table 1, and full quality ratings can be found in Supplementary Table 2.

All of the 29 studies employed a cross-sectional design and a range of neuroimaging modalities: DTI (*k* = 8), rsfMRI (*k* = 12), PET (*k* = 3), fMRI (*k* = 6), SPECT (*k* = 2) and MRS (*k* = 1). All studies included patients with idiopathic Parkinson’s disease with diagnosis mainly on the basis of the UK Parkinson’s Disease Society Brain Bank criteria.^58,59^ However, studies used a range of criteria to assess the presence of psychosis: clinical rating scales (e.g. the Neuropsychiatric Inventory, Movement Disorder Society Unified Parkinson’s Disease Rating Scale Part I Item 1.2, Scales for Outcomes in Parkinson’s Disease-psychiatric complications, Parkinson Psychosis Rating Scale, Bi-stable Percept Paradigm and the North–East Visual Hallucinations Interview) and the National Institute of Neurological Disorders and Stroke-National Institute of Mental Health criteria.^60^ The assessment of psychotic symptoms in Parkinson’s disease patients mainly focused on visual hallucinations (VHs). The most common cognitive outcome measure used in these studies was the mini-mental state examination.^61^

All 29 studies received a rating of 1 star for the ‘Selection’ item on the definition of PDP. Selection of PDnP patients was assigned one star in 28 studies, while the definition of such a group was clearly reported in 10 studies, which were assigned 1 star in this domain. ‘Comparability’ was good in all studies, i.e. patients were matched on age, gender and other clinical or demographic variables, while other studies reported these variables as covariates included in the analysis. Five studies received one star in this domain when only one factor, i.e. either age or another variable such as gender, was used as a covariate or to match patients. Only three studies had full scores on the ‘Exposure’ domain. Full quality ratings are reported in Supplementary Table 2.

### Task fMRI studies

**Figure 2:**
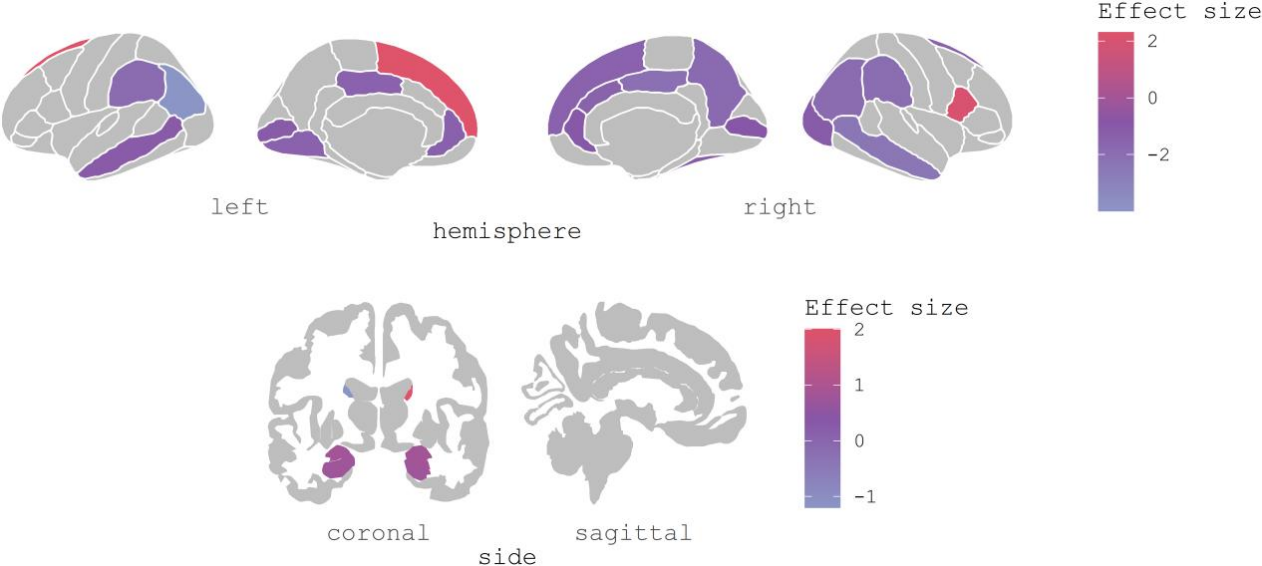
**Task-based fMRI studies brain plots.** Plots display the brain regions showing hyper-activation (pink) and hypo-activation (violet) across all fMRI studies (*k* = 6) in PDP patients (*n* = 80), where data were available. *Z*-Scores and/or *t*-values were extracted from the studies and converted into effect size estimates for purely display purposes (without carrying out any quantitative synthesis).

Six case–control cross-sectional studies employed fMRI in conjunction with cognitive activation paradigms such as signal detection,^22,57^ checkerboard paradigm,^48^ oddball paradigm to examine salience,^47^ apparent motion^23^ and one-back repetition detection task.^56^ These studies included a total of 174 patients, of which 80 were PDP patients (50% male, mean age ± SD = 67.80 ± 6.12 years, mean education ± SD = 9.39 ± 3.18 years) and 94 were PDnP patients (61.70% male, mean age ± SD = 68.07 ± 5.10 years, mean education ± SD = 9.15 ± 3.65 years). Patterns of cortical hyper-activation and hypo-activation were observed and were related to the task condition. In one study,^57^ before stimulus detection, PDP patients showed decreased activation in bilateral occipital cortex, superior frontal gyrus, bilateral fusiform gyrus and left lingual gyrus, cingulate cortex and right middle frontal gyrus compared with PDnP patients.^57^ While at the time of detection as reported by Lefebvre *et al*.,^22^ PDP patients showed increased activation in the right cerebellum, right pre-frontal cortex and right occipital cortex and hypo-activation of the left cingulate cortex, caudate nucleus, temporal and occipital cortices compared with PDnP patients (*n* = 16). In another study, Stebbins *et al*.^23^ applied moving (i.e. kinematic) and stroboscopic images to assess the functional correlates of visual perception of basic and apparent motion using a whole-brain analysis approach and reported greater activation in the left superior frontal gyrus in PDP (*n* = 12) compared with PDnP patients (*n* = 12). Conversely, PDnP patients reported greater activation of temporal–occipital lobe, supramarginal parietal lobe, inferior parietal lobe and cingulate cortex. Further small-volume correction showed additional reduced activation in V5/middle temporal visual area (MT) when kinematic stimuli were presented. In the same study, when stroboscopic images were presented, PDP patients had greater activation in the inferior frontal lobe and caudate nucleus and reduced activation in the cingulate cortex and inferior parietal lobe compared with PDnP patients. In contrast, using a different paradigm [i.e. checkerboard paradigm (previously described by Taylor *et al*.^62^)], Firbank *et al*.^48^ did not find any differences in V5 activation and in any other brain region between PDP and PDnP patients. Only one study examined the salience network. Knolle *et al*.^47^ examined salience processing by using an oddball visual task whereby patients were presented with alternating images of faces and outdoor scenes, which were either neutral or emotionally charged in terms of content (e.g. angry face or image of a car crash), and applied an ROI analysis approach focusing on four regions: substantia nigra (SN)/ventral tegmental area (VTA), ventral and dorsal striatum, bilateral hippocampus and bilateral amygdala. They observed greater activation of emotionally salient stimuli in PDP patients (*n* = 14) compared with PDnP patients (*n* = 23) in the amygdala, hippocampus, striatum and SN/VTA, but no evidence of difference in the opposite direction. Further, no differences in brain activation were observed between PDP and PDnP groups while processing novel but emotionally neutral stimuli.^47^ Using a one-back repetition detection task, Ramírez-Ruiz *et al*.^56^ showed hypo-activation upon signal detection in Parkinson’s disease patients with VH (*n* = 10) in the inferior, superior and middle frontal gyrus and anterior cingulate gyrus compared with PDnP patients (*n* = 10). However, greater activation in the right inferior frontal gyrus was reported in Parkinson’s disease patients with VH compared with PDnP patients during the control condition. Results are summarized visually in Fig. 2 and descriptively in Table 1.

**Table 1:** Qualitative summary of the main findings for each task-based fMRI study

Study (number of PDP and PDnP patients, sex and mean age)	Task details; analysis approach	Results
Firbank *et al*.^48^	Visual stimuli: checkboard paradigm; whole-brain and ROI approach (i.e. visual areas, V1, V2, V3, V4 and V5)	No differences between PDP and PDnP patients
PDP, *n* = 17 (13 M, 4 F); mean age: 75.5 years		
PDnP, *n* = 19 (17 M, 2 F); mean age: 72.3 years		
Knolle *et al*.^47^	Visual stimuli: odd ball for salience processing; ROI approach (i.e. SN/VTA, the ventral and dorsal striatum, bilateral hippocampus and bilateral amygdala)	Salient condition:
PDP, *n* = 14 (7 M, 7 F); mean age: 62.5 years		PDP: SN/VTA ↑, striatum ↑, hippocampus (bilateral) ↑ and amygdala (bilateral) ↑ compared with PDnP
PDnP, *n* = 23 (14 M, 9 F); mean age: 63.1 years		Novelty condition:
		No differences between PDP and PDnP patients
Lefebvre *et al*.^22^	Visual stimuli:^63^ visual perception task; whole-brain approach	Stimulus detection:
PDP, *n* = 18 (11 M, 7 F); mean age: 63.5 years		PDP: right cerebellum ↑, right occipital cortex ↑ and right pre-frontal cortex ↑ compared with PDnP
PDnP, *n* = 16 (12 M, 4 F); mean age: 62.7 years		PDP: left cingulate cortex ↓, left caudate nucleus ↓, left temporal ↓ and occipital cortices ↓ compared with PDnP patients
Stebbins *et al*.^23^	Visual stimuli: stroboscopic versus no visual stimulus, kinematic versus stationary visual; whole-brain approach	Stroboscopic stimulation:
PDP, *n* = 12 (NR); mean age: 71.2 years		PDP: right inferior frontal gyrus ↑ and right caudate nucleus ↑ compared with PDnP
		Kinematic stimulation:
PDnP, *n* = 12 (NR); mean age: 73.3 years		PDP: left superior frontal lobe ↑ compared with PDnP
Meppelink *et al*.^57^	Visual stimuli: detection task; whole-brain and ROI approach (as defined by the finite impulse response analysis)	Before stimulus detection:
PDP, *n* = 9 (5 M, 4 F); mean age: 61.2 years		PDP: bilateral occipital cortex ↓, superior frontal gyrus ↓, bilateral fusiform gyrus ↓, left lingual gyrus ↓, cingulate cortex ↓ and right middle frontal gyrus ↓ compared with PDnP
PDnP, *n* = 14 (11 M, 3 F); mean age: 64.6 years		
Ramírez-Ruiz *et al*.^56^	Visual stimuli: one-back repetition detection; whole-brain approach	Detection condition:
PDP, *n* = 10 (4 M, 6 F); mean age: 73.0 years		PDP: right inferior frontal gyrus ↓, bilateral superior frontal gyrus ↓, bilateral middle frontal gyrus ↓ and right anterior cingulate cortex ↓ compared with PDnP
PDnP, *n* = 10 (4 M, 6 F); mean age: 72.5 years		Control condition:
		PDP: right inferior frontal gyrus ↑ compared with PDnP

↓, hypo-activation; ↑, hyper-activation; F, female; M, male; NR, not reported.

### rsfMRI studies

Twelve studies employed rsfMRI to examine functional connectivity using different approaches. Two studies investigated functional connectivity within the default mode network (DMN),^13,39^ while four studies examined functional connectivity between hippocampus and whole brain^40^ and within and between multiple networks (i.e. attentional networks^21^ and the visual network,^46^ as well as effective connectivity within regions involved in bottom–up and top–down processing^44^). Three studies applied a seed-based approach to investigate functional connectivity between different nodes, specifically the posterior cingulate cortex (PCC), the superior medial frontal gyrus and the primary visual cortex, and whole brain,^43,45,51^ while two studies examined whole-brain functional connectivity,^53,54^ and one examined dynamic changes in functional connectivity.^52^ Tables 2 and 3 report a summary of these results. These studies included a total of 575 patients, of which 206 were PDP patients (49.03% male; mean age ± SD = 67.08 ± 3.71 years, mean education ± SD = 13.97 ± 4.22 years) and 369 were PDnP patients (57.96% male; mean age ± SD = 65.76 ± 2.71 years; mean education ± SD = 13.31 ± 4.39 years). In a sample of 32 PDP patients (18 with minor hallucinations), Bejr-Kasem *et al*.^13^ used the PCC as a seed ROI to investigate the co-activation at rest between the DMN and other attentional networks. In Parkinson’s disease patients with minor hallucinations, the PCC showed greater connectivity with bilateral middle temporal gyrus and bilateral superior parietal lobes, right precentral gyrus and left middle cingulate cortex, compared with PDnP patients. Further, they reported greater connectivity in Parkinson’s disease patients with minor hallucinations between PCC and left middle occipital and bilateral posterior middle temporal gyri compared with PDnP patients. These results did not change after controlling for PCC volume. A similar pattern of findings was also reported by Yao *et al*.^39^ who examined the DMN in 24 Parkinson’s disease patients, of whom 12 reported VH. With a sample of Parkinson’s disease patients with and without VH matched in terms of age, gender and cognitive abilities, Parkinson’s disease patients with VH reported greater functional connectivity in the right middle frontal gyrus and bilateral posterior cingulate gyrus, and precuneus was reported compared with PDnP patients. However, these functional connectivity alterations were not associated with the severity of psychotic symptoms. In another study,^43^ Parkinson’s disease patients with VH (*n* = 20) and Parkinson’s disease patients with visual illusions (VIs, *n* = 19) showed decreased functional connectivity between the left lingual gyrus and the left para-hippocampal region compared with PDnP (*n* = 23). Parkinson’s disease patients with VH also reported reduced connectivity between a seed cluster in the inferior occipital lobe and anterior cingulate, precuneus and para-hippocampal regions. In addition, Parkinson’s disease patients with VI reported greater functional connectivity compared with those with VH in the following regions: greater connectivity of the right hippocampal seed with left inferior frontal gyrus, left insula and right temporo-occipital fusiform cortex, right superior frontal seed with right inferior frontal gyrus and amygdala seed bilaterally with bilateral inferior frontal gyrus. In a sample of Parkinson’s disease patients with minor hallucinations (*n* = 23),^45^ Zhong *et al*.^45^ found increased connectivity between the bilateral medial superior frontal gyrus (SFGmed, i.e. used as seed) and left middle temporal gyrus, decreased connectivity between the SFGmed seed and right calcarine sulcus and left middle occipital gyrus compared with PDnP patients (*n* = 35), even after controlling for group differences in age, sex, education and neuropsychiatric symptoms (rapid eye movement [REM]) sleep behaviour disorder, anxiety and depression) and grey matter volume.^45^ Using spectral dynamic causal modelling, Thomas *et al*.^44^ found that Parkinson’s disease patients (with VH, *n* = 15) displayed decreased effective connectivity from the lateral geniculate nucleus to V1 and increased effective connectivity between pre-frontal cortices and V1 and medial thalamus compared with PDnP patients (*n* = 75). Shine *et al*.^21^ applied a bistable percept paradigm to define group allocation to Parkinson’s disease patients with VH (*n* = 10) or without VH (*n* = 9). Both Parkinson’s disease patients with and without VH underwent a visual task, which assessed the strength of mental imagery, and the total scores of this task were employed by the authors to investigate the relationship between the strength of mental imagery (i.e. the ability to recall a clear and vivid stimulus without the stimulus being physically present) and the co-activations of different attentional networks at rest. Clusters within the dorsal attention network, the DMN, the ventral attention network and the visual network were selected as ROIs. Stronger mental imagery was positively associated with the activation of the ventral attentional network and the DMN. Higher scores on the mental imagery task (suggestive of proneness to experience hallucinations) were associated with decreased connectivity between dorsal and ventral attentional networks and between dorsal attentional and visual networks. A similar pattern of results was reported by Diez-Cirarda *et al*.,^46^ who also investigated the visual network in PDP (*n* = 12) and PDnP patients (*n* = 35). PDP patients had decreased functional connectivity between the dorsal and ventral attentional network and between the ventral attentional network and the visual network and increased functional connectivity of the right frontal eye field (i.e. part of the dorsal attention network) with bilateral intra-parietal sulci compared with PDnP patients. In addition, Walpola *et al*.^51^ observed a relationship between mind-wandering and increased connectivity between primary visual network (V1), used as seed, and PCC, medial pre-frontal cortex, inferior parietal lobule, orbitofrontal cortex, inferior frontal gyrus and visual processing areas such as fusiform gyrus and inferior temporal gyrus in Parkinson’s disease with hallucinations (*n* = 18) compared with PDnP patients (*n* = 20). Using the hippocampus as a seed, Yao *et al*.^40^ examined hippocampal functional connectivity with the whole brain, and across analyses, the authors controlled for age, cognitive abilities (measured with the mini-mental state examination) and visual accuracy. PDnP patients (*n* = 15) showed reduced hippocampal functional connectivity with parietal and frontal regions. Conversely, Parkinson’s disease patients with VH (*n* = 12) reported reduced functional connectivity between the right hippocampus and bilateral cuneus and lingual gyrus, right fusiform gyrus, right medial temporal lobe and left superior/middle temporal gyrus; reduced functional connectivity between left hippocampus and bilateral lingual gyrus, right fusiform gyrus, left cuneus, right medial temporal lobe and right precuneus; and greater hippocampal functional connectivity with bilateral frontal lobe, cingulate cortex and inferior parietal lobe compared with PDnP patients. Zarkali *et al.*^52^ examined temporal dynamics by applying dynamic functional connectivity and network control theory in Parkinson’s disease with VH (*n* = 16). Network control theory integrates structural connectivity with linear assessments of local dynamics to simulate the brain shifts between multiple functional states.^64,65^ They reported altered temporal dynamics of functional connectivity compared with PDnP (*n* = 75), favouring a more functionally segregated state in Parkinson’s disease with VH. They also found that serotonergic (5-HT1_B_) and GABAergic (GABA_A_) (gamma-aminobutyric acid [GABA]) receptor density were negatively correlated with the transition between functional states based on network control theory in PDP patients with VH. Two studies did not report any difference in functional connectivity between PDP and PDnP.^53,54^

**Table 2:** Qualitative summary of the main findings for rsfMRI studies employing a seed-based analysis

Study (number of PDP and PDnP patients, sex and mean age)	Analytical approach; network(s) explored	Results
Bejr-Kasem *et al*.^13^	Seed-to-voxel (PCC as seed); whole-brain	PDP: ↑ PCC connectivity with bilateral middle temporal gyrus and bilateral superior parietal lobes, right precentral gyrus and left middle cingulate cortex compared with PDnP
PDP, *n* = 18 (10 M, 8 F); mean age: 70.4 years		
PDnP, *n* = 14 (10 M, 4 F); mean age: 65.8 years		
Marques *et al*.^43^	Seed-based connectivity; seed-to-voxel [seeds: (putamen, caudate, lateral occipital cortex, lingual gyrus and occipital, fusiform gyrus, amygdala and insula, hippocampus, precuneus and inferior/middle/superior frontal gyrus)]	PDP: ↓ functional connectivity between left lingual gyrus and left para-hippocampal region compared with PDnP
PDP, *n* = 19 (10 M, 9 F); mean age: 68.3 years		
PDnP, *n* = 23 (14 M, 9 F); mean age: 69.2 years		PDP with VI ↑ functional connectivity between right hippocampal seed with left inferior frontal gyrus, ↑ left insula with right temporo-occipital fusiform cortex, ↑ right superior frontal seed with right inferior frontal gyrus and ↑ bilateral amygdala with bilateral inferior frontal gyrus compared with PDP with VH
Zhong *et al*.^45^	Seed-to-voxel (SFGmed as seed); whole-brain	PDP: ↑ functional connectivity between SFGmed and left middle temporal gyrus compared with PDnP
PDP, *n* = 23 (6 M, 17 F); mean age: 60.6 years		
PDnP, *n* = 35 (13 M, 22 F); mean age: 61.1 years		PDP: ↓ functional connectivity between SFGmed and right calcarine sulcus, left middle occipital gyrus compared with PDnP
Yao *et al*.^40^	Seed-to-voxel; whole-brain, seed: hippocampus	PDP: ↓ functional co-activation between right hippocampus and bilateral cuneus and lingual gyrus, right fusiform, right medial temporal lobe and left superior/middle temporal gyrus compared with PDnP
PDP, *n* = 12 (10 M, 2 F); mean age: 69.4 years		PDP: ↓ functional connectivity between left hippocampus and bilateral lingual gyrus, right fusiform gyrus, left cuneus and right medial temporal lobe and right precuneus compared with PDnP
PDnP, *n* = 15 (10 M, 5 F); mean age: 66.3 years		PDP: ↑ hippocampal connectivity with bilateral frontal lobe, cingulate and inferior parietal lobe compared with PDnP
Walpola *et al*.^51^	Functional connectivity; inter-network and seed-to-voxel (primary visual network, V1, as seed)	PDP: strong association between ↑ mind-wandering and ↑ functional connectivity between V1, mPFC, IPL, PCC, orbitofrontal cortex, inferior frontal gyrus and fusiform/inferior temporal gyrus compared with PDnP
PDP, *n* = 18 (14 M, 4 F); mean age: 67.5 years		
PDnP, *n* = 20 (17 M, 3 F); mean age: 63.7 years		

F, female; M, male; mPFC, medial pre-frontal cortex; SFGmed, superior medial frontal gyrus.

**Table 3:** Qualitative summary of the main findings for rsfMRI studies employing different approaches to the analysis

Study (number of PDP and PDnP patients, sex and mean age)	Analytical approach; network(s) explored	Results
Yao *et al*.^39^	Probabilistic independent component analysis and dual regression; DMN	PDP: ↑ functional connectivity right superior middle frontal lobe and bilateral posterior cingulate gyrus/precuneus compared with PDnP
PDP, *n* = 12 (3 M, 9 F); mean age: 67.6 years		
PDnP, *n* = 12 (4 M, 8 F); mean age: 63.4 years		
Shine *et al*.^21^	Functional connectivity, ROI; attentional networks (AN, DAN, DMN) and visual network. ROI: bilateral superior parietal lobule, bilateral frontal eye field (DAN), midline precuneus, midline medial pre-frontal cortex, bilateral hippocampal formation (DMN), bilateral anterior insula, bilateral dorsal anterior cingulate cortex (VAN) and bilateral occipital cortex (visual network)	Mental imagery positively correlated with VAN and DMN
PDP, *n* = 10 (NR); mean age: 69.5 years		PDP: high mental imagery was related to ↓ functional connectivity between DAN and VAN, and DAN and visual network
PDnP, *n* = 9 (NR); mean age: 67.1 years		PDP: mental imagery was related to ↑ functional connectivity within VAN and DMN
Thomas *et al*.^44^	Spectral dynamic causal modelling	PDP: ↓ functional connectivity from LGN to V1 compared with PDnP
PDP, *n* = 15 (4 M, 11 F); mean age: 65.3 years		
PDnP, *n* = 75 (44 M, 31 F); mean age: 64.1 years		PDP: ↑ functional connectivity between left PFC and V1, and medial thalamus compared with PDnP
Diez-Cirarda *et al*.^46^	Functional connectivity; attentional networks (VAN, DAN and DMN) and visual network	PDP: ↓ functional connectivity between DAN and VAN and between VAN and visual network, compared with PDnP
PDP, *n* = 12 (5 M, 7 F); mean age: 59.0 years		
PDnP, *n* = 35 (22 M, 13 F); mean age: 63.5 years		PDP: ↑ functional connectivity with right frontal eye field and bilateral intra-parietal sulci compared with PDnP
Zarkali *et al*.^52^	Dynamic functional connectivity, network control theory; whole brain	PDP: spent more time in segregated states with fewer transitions between integrated and segregated states whole brain, with the top 20% nodes belonging to the visual, somatosensory cortices, VAN, limbic, fronto-parietal and DMN compared with PDnP
PDP, *n* = 16 (5 M, 11 F); mean age: 64.8 years		
PDnP, *n* = 75 (41 M, 34 F); mean age: 64.4 years		
Miloserdov *et al*.^53^	Independent component analysis; DMN, DAN, salience network, right fronto-parietal, somatomotor, visual medial and visual lateral cortex	Misperception scores were associated with ↓ functional connectivity between DAN and salience network
PDP, *n* = 16 (11 M, 5 F); mean age: 70.1 years PDnP, *n* = 16 (12 M, 4 F); mean age: 70.2 years		
		No differences between PDnP and PDP
Hepp *et al*.^54^	Functional connectivity; whole brain	No differences between PDP and PDnP patients
PDP, *n* = 15 (11 M, 4 F); mean age: 69.0 years		
PDnP, *n* = 40 (21 M, 19 F); mean age: 67.0 years		

↓, decrease; ↑, increase; DAN, dorsal attention network; F, female; IPL, inferior parietal lobule; LGN, lateral geniculate nucleus; M, male; mPFC, medial pre-frontal cortex; NR, not reported; PFC, pre-frontal cortex; SFGmed, superior medial frontal gyrus; VAN, ventral attention network.

### DTI studies

**Figure 3:**
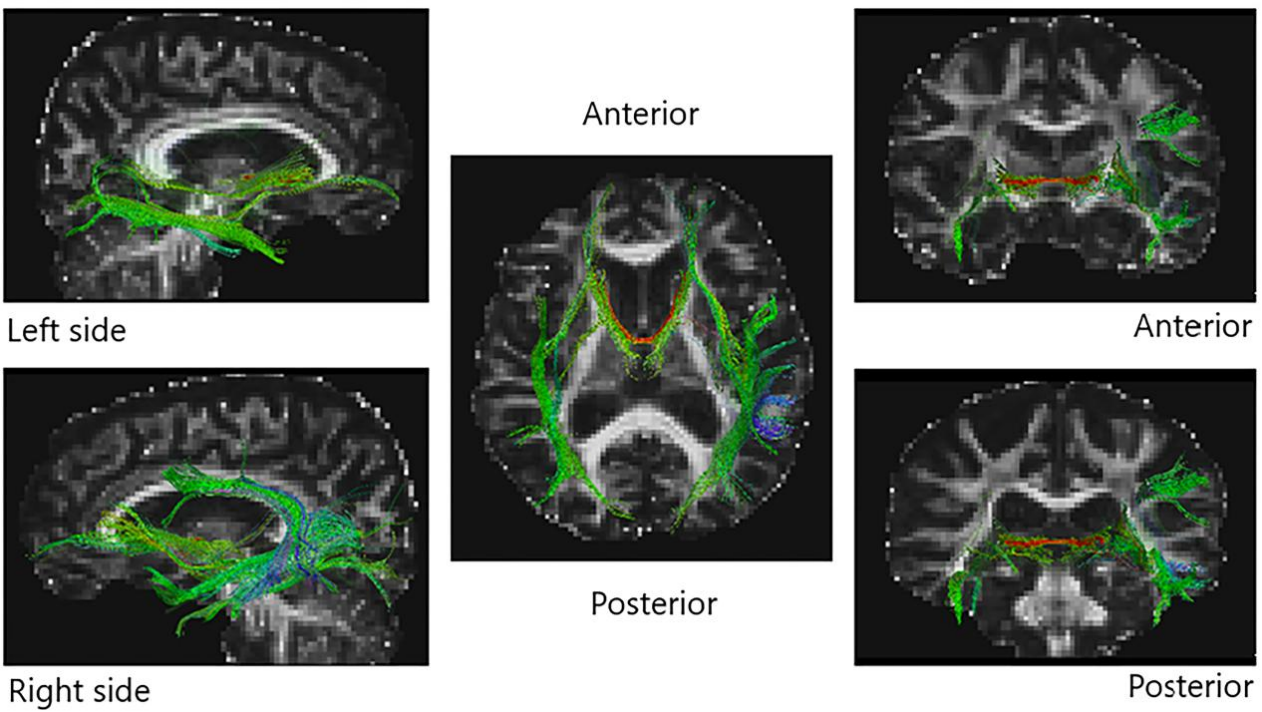
**Diffusion tensor imaging (DTI) studies brain plots.** White matter fibre tracts that were found dysfunctional in PDP patients compared to PDnP patients in DTI studies (*k*=8). Information was extracted from each paper and plotted on a brain template on Trackviz.^66^ These plots do not reflect any analyses conducted on these studies and are purely for display purposes.

Eight case–control studies used DTI to assess white matter tracts in PDP. Of 423 patients, 142 were PDP patients (63.71% male; mean age ± SD = 67.44 ± 5.28 years, mean education ± SD = 14.20 ± 4.10 years) and 281 were PDnP patients (56.68% male; mean age ± SD = 66.36 ± 6.01 years; mean education ± SD = 14.5 ± 3.39 years). Four studies applied an ROI approach,^26,40,42,50^ three applied TBSS^36,48,55^ and one used different analytical approaches such as anatomically constrained tractography and network-based statistics.^38^ Hepp *et al*.^26^ examined the nucleus basalis of Meynert (NBM), a cholinergic sub-structure located in the basal forebrain. They compared whole-brain fibre tracts between idiopathic Parkinson’s disease patients with VH (*n* = 15) and PDnP patients (*n* = 40), who were otherwise comparable in terms of demographic and clinical characteristics. No differences in mean diffusivity (MD) or FA between groups in tracts from the NBM and frontal, temporal, cingular and insular regions were observed. However, Parkinson’s disease patients with VH showed higher MD in parietal and occipital tracts compared with PDnP patients. Lee *et al*.^42^ investigated the lateral geniculate nucleus, the optic chiasma, optic radiation and optic nerve in age-matched and sex-matched Parkinson’s disease with VH (*n* = 10) and without VH (*n* = 14). Parkinson’s disease patients with VH showed alterations of all the regions investigated compared with PDnP patients. However, MD and axial diffusivity (AD) were significantly higher in the left optic radiation and right optic nerve in Parkinson’s disease patients with VH, respectively. The lateral geniculate nucleus showed mildly reduced volume in this patient group compared with PDnP patients. Only one study examined the hippocampus focusing on MD and voxel-by-voxel approach. Yao *et al*.^40^ observed higher MD in the right hippocampus and right posterior hippocampal regions in Parkinson’s disease patients with VH (*n* = 12) compared with PDnP patients (*n* = 15), even after accounting for differences in LEDD, sex and Parkinson’s disease severity. However, TBSS studies revealed an opposite pattern of results. Firbank *et al*.^48^ showed no significant differences in MD in PDP patients (*n* = 17) compared with PDnP patients (*n* = 18). Lee *et al*.^36^ reported widespread white matter abnormalities across the cortex, but these were not significantly different between patients (PDP, *n* = 10; PDnP, *n* = 21). In a sample of Parkinson’s disease with (*n* = 19) and without VH (*n* = 81), matched on age, sex, motor symptoms and cognitive abilities, Zarkali *et al*.^38^ revealed widespread structural connectivity dysfunctions in Parkinson’s disease patients with VH compared with PDnP patients. They applied network-based statistics and analysis of controllability, which enables estimation of the impact of cortical changes on brain functions. Their results showed a network of 82 nodes in Parkinson’s disease patients with VH that corresponds to regions involved in information processing and attention with reduced structural connectivity within this network in Parkinson’s disease patients with VH. Yuki *et al.*^50^ observed lower FA in PDP with VH (*n* = 17) in the left inferior longitudinal fasciculus (ILF) and in bilateral inferior fronto-occipital fasciculus (IFOF) compared with PDnP patients (*n* = 43). After adjusting for cognitive function (measured with the mini-mental state examination), they also reported that the presence of VH was associated with lower FA and higher MD in left ILF in PDP patients. The same findings were reported using TBSS by Lenka *et al*.,^55^ who also reported lower FA in the corpus callosum, right ILF, right occipito-parietal white matter tracts and corticospinal tracts in PDP (*n* = 42) compared with PDnP patients (*n* = 48; Fig. 3, Table 4).

**Table 4:** Qualitative summary of the main findings for each DTI study

Study (number of PDP and PDnP patients, sex and mean age)	Analytical approach; areas of interest	Results
Lee *et al*.^36^	TBSS, FA, MD; whole brain	PDP: ↓ FA in both fronto-temporo-parietal and brainstem regions compared with PDnP. ↑ MD values in left temporo-parietal and lateral geniculate areas
PDP, *n* = 10 (7 M, 3 F); mean age: 69.4 years		
PDnP, *n* = 21 (9 M, 11 F); mean age: 66.2 years		
Yuki *et al*.^50^	FA, MD; ROI: ILF, IFOF	PDP: ↓ FA and ↑ MD values in left ILF compared with PDnP
PDP, *n* = 17 (6 M, 11 F); mean age: 73.9 years		
PDnP, *n* = 43 (17 M, 26 F); mean age: 76.6 years		
Firbank *et al*.^48^	TBSS, FA, MD; whole brain	No differences between PDP and PDnP
PDP, *n* = 17 (13 M, 4 F); mean age: 75.5 years		
PDnP, *n* = 19 (17 M, 2 F); mean age: 72.3 years		
Lenka *et al*.^55^	TBSS, FA, MD, AD, RD; whole brain	PDP: ↓ FA in two clusters including (i) corpus callosum, right ILF, corticospinal tract and right occipito-parietal WM and (ii) left ILF and IFOF compared with PDnP
PDP, *n* = 42 (35 M, 7 F); mean age: 58.5 years		
PDnP, *n* = 48 (41 M, 7 F); mean age: 57.9 years		No differences between groups on MD, AD and RD
Lee *et al*.^42^	FA, MD, AD, RD; ROI: ON, OC, LGN, OR	PDP: ↓ FA in left ON and smaller LGN, ↑ MD and ↑ AD values in left OR compared with PDnP
PDP, *n* = 10 (7 M, 3 F); mean age: 69.2 years		
PDnP, *n* = 14 (5 M, 9 F); mean age: 66.1 years		
Yao *et al*.^40^	MD, voxel-by-voxel analysis; ROI: hippocampus	PDP: ↑ MD in right hippocampus compared with PDnP. There was also ↑ MD in posterior hippocampal areas compared with PDnP
PDP, *n* = 12 (10 M, 2 F); mean age: 69.4 years		
PDnP, *n* = 15 (10 M, 5 F); mean age: 66.3 years		
Hepp *et al*.^26^	FA, MD; NBM	PDP: ↑ MD values in pathways connecting the NBM to the cerebral cortex and in parietal and occipital tracts compared with PDnP
PDP, *n* = 15 (11 M, 4 F); mean age: 69.0 years		
PDnP, *n* = 40 (21 M, 19 F); mean age: 67.0 years		No differences on MD between PDP and PDnP
Zarkali *et al*.^38^	Network-based statistics; whole brain	PDP: clusters of ↓ connectivity compared with PDnP
PDP, *n* = 19 (6 M, 13 F); mean age: 64.6 years		
PDnP, *n* = 81 (47 M, 34 F); mean age: 64.4 years		

↓, decrease; ↑, increase; F, female; IFOF, inferior fronto-occipital fasciculus; LGN, lateral geniculate nucleus; M, male; OC, optic chiasm; ON, optic nerve; OR, optic radiation; RD, radial diffusivity; WM, white matter.

### PET studies

**Figure 4:**
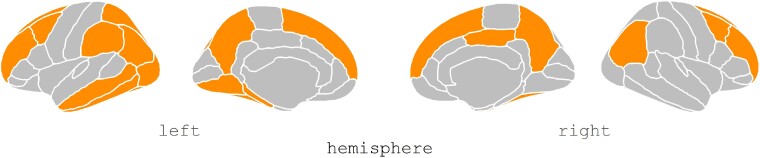
**PET studies brain plots.** Plot shows brain regions with hypo-metabolism (ROIs are highlighted in orange) in PDP patients (*n* = 75) compared with PDnP patients (*n* = 77). All figures were created using *ggseg*^31^ in R.

Three case–control studies employed PET imaging to examine glucose metabolism in PDP patients. A total of 152 patients, of which 75 were PD with VH (50.6% males; mean age ± SD = 69.52 ± 2.40 years; mean education ± SD = 12.05 ± 1.99 years) and 77 were PDnP patients (49.4% males; mean age ± SD = 67.52 ± 2.65 years; mean education ± SD = 10.20 ± 1.41 years), were analysed, and all studies employed an 18-F-FDG PET. In one study with Parkinson’s disease patients with different psychotic symptoms,^41^ the authors employed partial least square correlation analysis to compare glucose metabolism between Parkinson’s disease patients with VH (*n* = 19), VI (*n* = 17), kinetopsia (*n* = 24) (i.e. misperception of stationary objects as moving) and Parkinson’s disease without any such symptoms. The presence of VH and kinetopsia was associated with hypo-metabolism in temporo-parietal regions, while VI was associated with hypo-metabolism in occipital and temporo-parietal metabolism. However, patients across groups differed in socio-demographics and other clinical variables, i.e. Parkinson’s disease with VH were older and with greater cognitive impairment, motor symptoms and higher amount of LEDD compared with PDnP. One early study also supports this finding. Boecker *et al*.^24^ examined Parkinson’s disease patients with VH (*n* = 8) and PDnP patients (*n* = 11), who were matched on age, gender and LEDD. They found reduced metabolism in the left inferior parietal lobule, left supramarginal gyrus, bilateral precuneus, right inferior parietal lobule, right cingulate cortex, left middle frontal gyrus and left middle temporal gyrus in Parkinson’s disease patients with VH (corrected for false discovery rate) compared with PDnP patients. Left para-hippocampal gyrus and left lingual gyrus (both associated with ventral visual pathway) were also found to have a low glucose consumption rate in Parkinson’s disease with VH. A third study also examined cognitive impairments in Parkinson’s disease patients with VH (*n* = 15) compared with PDnP patients (*n* = 13).^37^ Parkinson’s disease patients with VH showed hypo-metabolism bilaterally in the middle and inferior temporal cortices, left lingual gyrus and left angular gyrus compared with PDnP patients. Parkinson’s disease patients with VH and cognitive impairment further showed reduced metabolism in middle and inferior temporal cortices, bilateral frontal areas, left superior parietal and occipital regions (uncorrected, *P* < 0.01), in the precuneus, bilaterally and superior parietal areas as well as in frontal, temporal and occipital lobes (uncorrected, *P* < 0.001) compared with those without cognitive impairments. Overall, PDP patients exhibited reduced metabolism in occipito-temporo-parietal regions, including areas within the dorsal and ventral visual pathways, and in bilateral frontal, temporal and occipital cortices compared with PDnP patients, irrespective of the presence or absence of mild cognitive impairment (Fig. 4).

### SPECT studies

**Figure 5:**
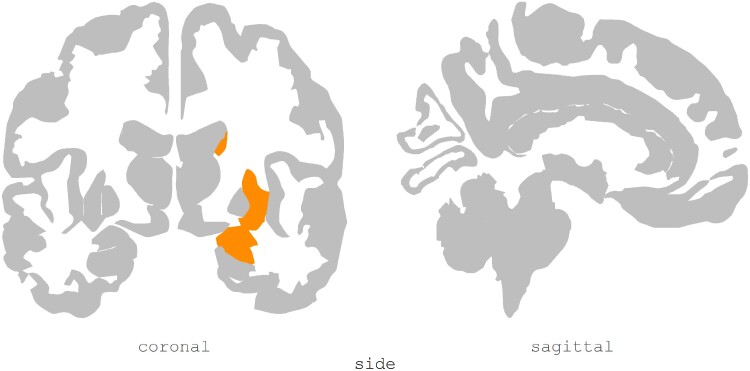
**SPECT studies brain plots.** Pattern of abnormal DAT binding in PDP patients (*n* = 40). The region highlighted in orange represents the regions with significantly different DAT binding ratios between PDP and PDnP patients, and this graph was done purely for display purposes.

Only two studies employed a [^123^ I] FP-CIT SPECT scan, which is applied to examine dopamine transporter (DAT) availability in specific brain regions (PDP, *n* = 40; PDnP, *n* = 66). Jaakkola *et al*.^49^ examined longitudinally a cohort of Parkinson’s disease patients who then developed psychotic symptoms. Although there was no difference in socio-demographics and in Parkinson’s disease medications between patients who transitioned to PDP, their results showed lower DAT binding at baseline in the right and left ventral striatum and right putamen compared with those who never developed psychosis. They also measured the binding ratio in specific ROIs: bilateral caudate, bilateral anterior putamen and bilateral posterior putamen. They observed that decreasing binding ratio in the ventral striatum bilaterally and right putamen in PDP patients compared with PDnP patients and reported asymmetry in putamen in the former. At follow-up, PDP patients had reduced DAT binding in the right amygdala compared with PDnP patients. Similarly, Kiferle *et al*.^35^ retrospectively analysed data from Parkinson’s disease patients (PDP, *n* = 36) who then developed psychosis (PDnP, *n* = 18) at 72.8 months from Parkinson’s disease onset (61.5 months after starting Parkinson’s disease medications). Reductions in DAT binding in the right caudate in PDP patients were reported at baseline compared with PDnP patients; the two groups did not differ in cognitive abilities, Parkinson’s disease symptoms and LEDD. Overall, PDP patients reported reduced DAT binding in the right caudate at baseline,^35^ lower DAT binding in right and left ventral striatum and right putamen and decreased binding ratio in ventral striatum bilaterally and right putamen^49^ compared with PDnP patients (Fig. 5).

### MRS study

Only one study included MRS as part of their examination in conjunction with fMRI and DTI approaches.^48^ They investigated GABA signal, which is indicative of the concentration of metabolic GABA, and levels of ambient extracellular GABA and observed reduced GABA+/creatine in PD with VH compared with PDnP patients, which was independent of cognitive functions (measured with the Cambridge Cognitive battery). No other studies employing the MRS technique were found in the search.

## Discussion

Here, we synthesized evidence from neuroimaging studies across multiple modalities using a qualitative approach with a view to identify key brain alterations that may be implicated in PDP. Findings from task-based fMRI studies revealed differential patterns of hyper-activation and hypo-activation across the cerebral cortex in PDP patients, and these were mainly localized in temporal, parietal and occipital lobes. Some areas of hyper-activation were observed in the frontal lobe but few studies reported such increased activation.^22,47^ Alterations in brain activity found in task-based fMRI studies varied according to the cognitive paradigm employed in the studies. While no consistent pattern of functional alteration was observed across the different cognitive paradigms, this may reflect both differences in cognitive processes engaged by these paradigms as well as analytic approaches that included whole-brain or ROI analysis approaches. Evidence from rsfMRI studies more commonly reported patterns of increased connectivity of frontal, parietal and temporal areas in PDP patients. Although employing different analytical approaches (e.g. seed-to-voxel, ROI, dynamic functional connectivity), these results showed increased connectivity at rest across areas primarily encompassing attentional networks. Using different seeds, e.g. PCC, bilateral hippocampus and the DMN, frontal regions appear to show greater functional connectivity with these seeds in PDP compared with PDnP patients. Results from DTI studies present evidence, which suggests additional types of connection abnormality in PDP patients, that is, microstructural damage and white matter abnormalities, with consistent results showing higher MD and lower FA in occipital, parietal tracts and areas involved in visual processing^26,42^ as well as dysfunction in hippocampal tracts.^40^ However, two DTI studies reported no difference between patient groups.^36,48^ Consistent patterns of hypo-metabolism were reported in PDP across all studies (*k* = 3) in occipito-temporo-parietal areas as well as frontal cortices when cognitive impairments were controlled for in the analysis. Reduced DAT binding in sub-cortical regions in PDP was reported in both SPECT studies, and it is in line with previous research.^67^ Dave *et al*.^67^ have also found that incidence of psychotic symptoms was associated with reduced striatal DAT binding ratio at baseline. Collectively, these findings suggest a widespread network of functional, metabolic and white matter dysfunctions in PDP patients. These were evident primarily in occipito-parietal-temporal regions in PDP compared with PDnP patients, thus in agreement with previous evidence.^8,28,55,68^ These areas are part of the dorsal and ventral visual pathways, involved in higher order visual processing, which may suggest a more prominent role of visual information processing in the manifestation of PDP. Current models suggest PDP arises from dysfunctional activation patterns between bottom–up and top–down processes as well as altered connections within different attentional networks.^69^ Here, we also observed dysfunctions in nodes within the DMN and attentional networks, which further aligns with previous evidence suggesting a possible over-reliance on self-referential and internal endogenous processes, due to disengagement or abnormal functioning of dorsal and ventral attentional networks giving rise to hallucinations in Parkinson’s disease patients.^20,44,46,70-75^ Although occipital, temporal and parietal areas appear to be most affected in PDP patients, evidence from PET and functional studies (both task-based and rsfMRI) also revealed patterns of dysfunction in frontal and sub-cortical cortices (i.e. frontal gyri, cingulate cortex). These areas are involved in executive and cognitive functions, such as selective attention, cognitive flexibility, inhibition (frontal cortices),^76,77^ internally directed thoughts, arousal, cognitive control and emotional processing (cingulate cortex).^78-80^ Abnormalities within these areas may further contribute to the rise of psychotic symptoms in Parkinson’s disease and may indicate that a wider cortical network involved in sophisticated executive functions may be also responsible for these symptoms. Furthermore, evidence from DTI and rsfMRI studies here reviewed appear to also suggest a possible role of the hippocampus (both as seed of interest and as part of the DMN) in PDP showing microstructural damages in the posterior hippocampus and abnormal patterns of functional co-activation in bilateral hippocampi^21,40^ with parietal, temporal and frontal areas. This is also aligned with the findings from the mega-analysis by Vignando *et al*.,^17^ which reported grey matter volume reduction in the hippocampus bilaterally, after covarying for age, gender, disease onset, Parkinson’s disease severity, medication and cognition, suggesting a role of this area independent of cognitive decline in PDP. This may suggest that the development of psychosis in Parkinson’s disease patients may be due to cortical volume loss in certain nodes, e.g. hippocampus, consequently leading to abnormal functionality at a network level. However, more research is needed to understand the potential temporal relationship between cortical volume loss and abnormal activation across the cortex. Future studies should also be carefully tailored to investigate the role of the hippocampus in this clinical population. Integrating innovative analytical approaches, such as combining structural and functional neuroimaging techniques with gene expression analysis and dynamic causal modelling (such as the work done by Zarkali *et al*.^52^), could offer a more comprehensive framework for understanding PDP.

## Limitations and strengths

We were unable to quantitatively synthesize data from different neuroimaging modalities due to the numerous statistical methodologies employed, e.g. ROI versus Whole brain, seed-to-voxel versus network analysis and the limited number of studies using PET and SPECT. Therefore, drawing definitive conclusions from these findings may be challenging. Similarly for DTI studies, we were unable to meaningfully analyse their results due to different techniques employed, e.g. TBSS^36,48,55^ versus ROI^26,40,42^ versus network-based statistics.^38^ We were also unable to relate patterns of abnormal activity or connectivity with different types of psychotic symptoms as an insufficient number of studies reported these. Future studies need to examine the association between the wide spectrum of psychosis manifestations in Parkinson’s disease, such as presence/passage, auditory and multi-modal hallucinations as well as delusions, to shed further light on common pathways shared by different types of symptoms. In addition, we noticed a prominent use of visual tasks in task-based fMRI studies specifically targeting general visual information processing. One study^47^ used a visual task in the context of emotional salience, which suggested the role of sub-cortical areas in processing emotionally charged images in PDP. Further studies should consider employing a variety of fMRI tasks to examine for example the activation of memory-related areas (e.g. hippocampus) and other cognitive domains (e.g. working memory, executive functions). These can further expand current knowledge on the neural correlates contributing to the development of psychosis in Parkinson’s disease patients. We only found one study using the MRS approach in this clinical population. This is quite interesting. MRS modality can provide helpful information on the local concentration of specific chemicals (e.g. glutamate) in specific cortical and sub-cortical areas. Such a modality can shed further light on the neurobiological mechanisms possibly involved in PDP. Last, this is a systematic synthesis of different studies and therefore aimed to provide a qualitative framework to better understand the neural substrates implicated in PDP. We attempted to synthesize the results from different studies using graphical representations of the brain areas involved in PDP by employing *ggseg* package.^31^ This enabled us to visually represent patterns of abnormal activity and metabolism and connectivity in PDP patients compared with PDnP patients. We also employed Trackviz^66^ to visualize fibre tracts that were dysfunctional in PDP patients. This can provide initial evidence to conduct future meta-analysis using a quantitative approach to examine each neuroimaging modality.

## Conclusion

The qualitative synthesis revealed extensive dysfunction in PDP patients, ranging from hypo-activation in task-based fMRI and abnormal functional connectivity in rsfMRI studies, hypo-metabolism and microstructural damage in this clinical population. There is also initial evidence suggesting a role of parietal, temporal and occipital regions involved in higher order visual processing and endogenous/exogenous attentional networks, as well as sub-cortical areas, such as the hippocampus. These findings may reflect the preponderance of Parkinson’s disease patients with VHs investigated in these studies.

## Supplementary Material

fcae358_Supplementary_Data

## Data Availability

This publication is a systematic review, and as such, it includes data that have already been published. The data that support the findings of this study are available from the corresponding author upon request.
