# Supplementary material for: Functional and connectivity correlates associated with Parkinson’s disease psychosis: a systematic review
Source: Brain Commun. 2024 Nov 6;6(6):fcae358. doi: 10.1093/braincomms/fcae358 (PMC11538965; doi:10.1093/braincomms/fcae358)
Supplement: fcae358_Supplementary_Data [file fcae358_supplementary_data.pdf]

Supplementary material

Sara Pisani, Brandon Gunasekera, Yining Lu, Miriam Vignando, Dominic ffytche, Dag Aarsland, K. Ray Chaudhuri, Clive Ballard, Jee-Young Lee, Yu Kyeong Kim, Latha Velayudhan, Sagnik Bhattacharyya.

Functional and connectivity correlates associated with Parkinson's Disease psychosis: A systematic review

Contents

Supplementary Material 2

Search strategy 2

Supplementary Table 1 11

Supplementary Table 2 18

Supplementary Material

Search strategy

Search strategy conducted on 25th January 2023 on Embase (Ovid)

1. Brain.mp. [mp=title, abstract, heading word, drug trade name, original title, device manufacturer, drug manufacturer, device trade name, keyword, floating subheading word, candidate term word]

2. Brain region*.mp. [mp=title, abstract, heading word, drug trade name, original title, device manufacturer, drug manufacturer, device trade name, keyword, floating subheading word, candidate term word]

3. Brain activit*.mp. [mp=title, abstract, heading word, drug trade name, original title, device manufacturer, drug manufacturer, device trade name, keyword, floating subheading word, candidate term word]

4. Functional connect*.mp. [mp=title, abstract, heading word, drug trade name, original title, device manufacturer, drug manufacturer, device trade name, keyword, floating subheading word, candidate term word]

5. Functional imaging.mp. [mp=title, abstract, heading word, drug trade name, original title, device manufacturer, drug manufacturer, device trade name, keyword, floating subheading word, candidate term word]

6. Neurophysiological mechanism*.mp. [mp=title, abstract, heading word, drug trade name, original title, device manufacturer, drug manufacturer, device trade name, keyword, floating subheading word, candidate term word]

7. Neuroanatomical correlate*.mp. [mp=title, abstract, heading word, drug trade name, original title, device manufacturer, drug manufacturer, device trade name, keyword, floating subheading word, candidate term word]

8. Neural substrate*.mp. [mp=title, abstract, heading word, drug trade name, original title, device manufacturer, drug manufacturer, device trade name, keyword, floating subheading word, candidate term word]

9. Neural correlate*.mp. [mp=title, abstract, heading word, drug trade name, original title, device manufacturer, drug manufacturer, device trade name, keyword, floating subheading word, candidate term word]

10. Cerebral mechanism*.mp. [mp=title, abstract, heading word, drug trade name, original title, device manufacturer, drug manufacturer, device trade name, keyword, floating subheading word, candidate term word]

11. Cerebral atrophy.mp. [mp=title, abstract, heading word, drug trade name, original title, device manufacturer, drug manufacturer, device trade name, keyword, floating subheading word, candidate term word]

12. Whole brain analys*.mp. [mp=title, abstract, heading word, drug trade name, original title, device manufacturer, drug manufacturer, device trade name, keyword, floating subheading word, candidate term word]

13. "MRI".mp. [mp=title, abstract, heading word, drug trade name, original title, device manufacturer, drug manufacturer, device trade name, keyword, floating subheading word, candidate term word]

14. Magnetic resonance imaging.mp. [mp=title, abstract, heading word, drug trade name, original title, device manufacturer, drug manufacturer, device trade name, keyword, floating subheading word, candidate term word]

15. Structural MRI.mp. [mp=title, abstract, heading word, drug trade name, original title, device manufacturer, drug manufacturer, device trade name, keyword, floating subheading word, candidate term word]

16. Structural magnetic resonance imaging.mp. [mp=title, abstract, heading word, drug trade name, original title, device manufacturer, drug manufacturer, device trade name, keyword, floating subheading word, candidate term word]

17. "fMRI".mp. [mp=title, abstract, heading word, drug trade name, original title, device manufacturer, drug manufacturer, device trade name, keyword, floating subheading word, candidate term word]

18. Functional MRI.mp. [mp=title, abstract, heading word, drug trade name, original title, device manufacturer, drug manufacturer, device trade name, keyword, floating subheading word, candidate term word]

19. Functional magnetic resonance imaging.mp. [mp=title, abstract, heading word, drug trade name, original title, device manufacturer, drug manufacturer, device trade name, keyword, floating subheading word, candidate term word]

20. Resting state fMRI.mp. [mp=title, abstract, heading word, drug trade name, original title, device manufacturer, drug manufacturer, device trade name, keyword, floating subheading word, candidate term word]

21. "rsfMRI".mp. [mp=title, abstract, heading word, drug trade name, original title, device manufacturer, drug manufacturer, device trade name, keyword, floating subheading word, candidate term word]

22. tractography.mp. [mp=title, abstract, heading word, drug trade name, original title, device manufacturer, drug manufacturer, device trade name, keyword, floating subheading word, candidate term word]

23. Diffusion tensor imaging.mp. [mp=title, abstract, heading word, drug trade name, original title, device manufacturer, drug manufacturer, device trade name, keyword, floating subheading word, candidate term word]

24. "DTI".mp. [mp=title, abstract, heading word, drug trade name, original title, device manufacturer, drug manufacturer, device trade name, keyword, floating subheading word, candidate term word]

25. Positron emission tomography.mp. [mp=title, abstract, heading word, drug trade name, original title, device manufacturer, drug manufacturer, device trade name, keyword, floating subheading word, candidate term word]

26. "PET".mp. [mp=title, abstract, heading word, drug trade name, original title, device manufacturer, drug manufacturer, device trade name, keyword, floating subheading word, candidate term word]

27. "SPECT".mp. [mp=title, abstract, heading word, drug trade name, original title, device manufacturer, drug manufacturer, device trade name, keyword, floating subheading word, candidate term word]

28. Single photon emission computed tomography.mp. [mp=title, abstract, heading word, drug trade name, original title, device manufacturer, drug manufacturer, device trade name, keyword, floating subheading word, candidate term word]

29. arterial spin labelling.mp. [mp=title, abstract, heading word, drug trade name, original title, device manufacturer, drug manufacturer, device trade name, keyword, floating subheading word, candidate term word]

30. Voxel-base*.mp. [mp=title, abstract, heading word, drug trade name, original title, device manufacturer, drug manufacturer, device trade name, keyword, floating subheading word, candidate term word]

31. Voxel-base morphometry.mp. [mp=title, abstract, heading word, drug trade name, original title, device manufacturer, drug manufacturer, device trade name, keyword, floating subheading word, candidate term word]

32. "VBM".mp. [mp=title, abstract, heading word, drug trade name, original title, device manufacturer, drug manufacturer, device trade name, keyword, floating subheading word, candidate term word]

33. Magnetic resonance spectroscopy.mp. [mp=title, abstract, heading word, drug trade name, original title, device manufacturer, drug manufacturer, device trade name, keyword, floating subheading word, candidate term word]

34. "MRS".mp. [mp=title, abstract, heading word, drug trade name, original title, device manufacturer, drug manufacturer, device trade name, keyword, floating subheading word, candidate term word]

35. Neuroimaging.mp. [mp=title, abstract, heading word, drug trade name, original title, device manufacturer, drug manufacturer, device trade name, keyword, floating subheading word, candidate term word]

36. 1 or 2 or 3 or 4 or 5 or 6 or 7 or 8 or 9 or 10 or 11 or 12 or 13 or 14 or 15 or 16 or 17 or 18 or 19 or 20 or 21 or 22 or 23 or 24 or 25 or 26 or 27 or 28 or 29 or 30 or 31 or 32 or 33 or 34 or 35

37. Parkinson's disease psychosis.mp. [mp=title, abstract, heading word, drug trade name, original title, device manufacturer, drug manufacturer, device trade name, keyword, floating subheading word, candidate term word]

38. Parkinson disease psychosis.mp. [mp=title, abstract, heading word, drug trade name, original title, device manufacturer, drug manufacturer, device trade name, keyword, floating subheading word, candidate term word]

39. 37 or 38

40. exp Parkinson disease/

41. Parkinson*.mp. [mp=title, abstract, heading word, drug trade name, original title, device manufacturer, drug manufacturer, device trade name, keyword, floating subheading word, candidate term word]

42. Parkinsonian.mp. [mp=title, abstract, heading word, drug trade name, original title, device manufacturer, drug manufacturer, device trade name, keyword, floating subheading word, candidate term word]

43. Parkinsonism.mp. [mp=title, abstract, heading word, drug trade name, original title, device manufacturer, drug manufacturer, device trade name, keyword, floating subheading word, candidate term word]

44. Atypical parkinsonism.mp. [mp=title, abstract, heading word, drug trade name, original title, device manufacturer, drug manufacturer, device trade name, keyword, floating subheading word, candidate term word]

45. 40 or 41 or 42 or 43 or 44

46. exp psychosis/

47. Psychotic.mp. [mp=title, abstract, heading word, drug trade name, original title, device manufacturer, drug manufacturer, device trade name, keyword, floating subheading word, candidate term word]

48. Psychotic disorder*.mp. [mp=title, abstract, heading word, drug trade name, original title, device manufacturer, drug manufacturer, device trade name, keyword, floating subheading word, candidate term word]

49. Paranoia.mp. [mp=title, abstract, heading word, drug trade name, original title, device manufacturer, drug manufacturer, device trade name, keyword, floating subheading word, candidate term word]

50. Paranoid.mp. [mp=title, abstract, heading word, drug trade name, original title, device manufacturer, drug manufacturer, device trade name, keyword, floating subheading word, candidate term word]

51. Delusi*.mp. [mp=title, abstract, heading word, drug trade name, original title, device manufacturer, drug manufacturer, device trade name, keyword, floating subheading word, candidate term word]

52. Halluci*.mp. [mp=title, abstract, heading word, drug trade name, original title, device manufacturer, drug manufacturer, device trade name, keyword, floating subheading word, candidate term word]

53. Visual halluci*.mp. [mp=title, abstract, heading word, drug trade name, original title, device manufacturer, drug manufacturer, device trade name, keyword, floating subheading word, candidate term word]

54. Auditory halluci*.mp. [mp=title, abstract, heading word, drug trade name, original title, device manufacturer, drug manufacturer, device trade name, keyword, floating subheading word, candidate term word]

55. multimodal halluci*.mp. [mp=title, abstract, heading word, drug trade name, original title, device manufacturer, drug manufacturer, device trade name, keyword, floating subheading word, candidate term word]

56. Visual illusion*.mp. [mp=title, abstract, heading word, drug trade name, original title, device manufacturer, drug manufacturer, device trade name, keyword, floating subheading word, candidate term word]

57. Illusion*.mp. [mp=title, abstract, heading word, drug trade name, original title, device manufacturer, drug manufacturer, device trade name, keyword, floating subheading word, candidate term word]

58. Imagery.mp. [mp=title, abstract, heading word, drug trade name, original title, device manufacturer, drug manufacturer, device trade name, keyword, floating subheading word, candidate term word]

59. Schizophrenia spectrum disorder*.mp. [mp=title, abstract, heading word, drug trade name, original title, device manufacturer, drug manufacturer, device trade name, keyword, floating subheading word, candidate term word]

60. Psychosis spectrum disorder*.mp. [mp=title, abstract, heading word, drug trade name, original title, device manufacturer, drug manufacturer, device trade name, keyword, floating subheading word, candidate term word]

61. 46 or 47 or 48 or 49 or 50 or 51 or 52 or 53 or 54 or 55 or 56 or 57 or 58 or 59 or 60

62. 45 and 61

63. 39 or 62

64. 36 and 63

Results, N = 6,608

Search strategy conducted on 25th January 2023 on PubMed

Search number	Query	Sort By	Filters	Results
7	(#1) AND (#6)			1,436
6	(#2) OR (#5)			3,782
5	(#3) AND (#4)			3,417
4	((((((((((((((Psychosis[MeSH Terms]) OR (Psychotic)) OR (Psychotic disorder*)) OR (Paranoia)) OR (Paranoid)) OR (Delusi*)) OR (Halluci*)) OR (Visual halluci*)) OR (Auditory halluci*)) OR (Multimodal halluci*)) OR (Visual illusion*)) OR (Illusion*)) OR (Imagery)) OR (Schizophrenia spectrum disorder*)) OR (Psychosis spectrum disorder*)			119,525
3	((((Parkinson disease[MeSH Terms]) OR (Parkinson*)) OR (Parkinsonian)) OR (Parkinsonism)) OR (Atypical Parkinsonism)			103,845
2	(Parkinson's disease psychosis) OR (Parkinson disease psychosis)			1,513
1	((((((((((((((((((((((((((((((((Brain) OR (Brain region*)) OR (Brain activit*)) OR (Functional connect*)) OR (Functional imaging)) OR (Neurophysiological mechanism*)) OR (Neuroanatomical correlate*)) OR (Neural substrate*)) OR (Neural correlate*)) OR (Cerebral mechanism*)) OR (Cerebral atrophy)) OR (Whole brain analys*)) OR (MRI)) OR (Magnetic resonance imaging)) OR (Structural MRI)) OR (Structural magnetic resonance imaging)) OR (fMRI)) OR (Functional MRI)) OR (Functional magnetic resonance imaging)) OR (Resting state fMRI)) OR (rsfMRI)) OR (tractography)) OR (Diffusion tensor imaging)) OR (DTI)) OR (Positron emission tomorgraphy)) OR (PET)) OR (SPECT)) OR (single photon emission computed tomography)) OR (arterial spin labelling)) OR (Voxel-base*)) OR (Voxel-base morphometry)) OR (VBM)) OR (magnetic resonance spectroscopy)) OR (MRS)) OR (Neuroimaging)			2,034,224

Search strategy conducted on 25th January 2023 on Web of Science

Set	Results	Save History / Create AlertOpen Saved History

# 10	370	**#7 AND #9**<br><br>*Indexes=SCI-EXPANDED, SSCI, A&HCI, CPCI-S, CPCI-SSH, ESCI Timespan=All years*
# 9	2,833	#8 OR #4<br><br>*Indexes=SCI-EXPANDED, SSCI, A&HCI, CPCI-S, CPCI-SSH, ESCI Timespan=All years*
# 8	8,537	#6 AND #5<br><br>*Indexes=SCI-EXPANDED, SSCI, A&HCI, CPCI-S, CPCI-SSH, ESCI Timespan=All years*
# 7	67,438	#3 AND #2 AND #1<br><br>*Indexes=SCI-EXPANDED, SSCI, A&HCI, CPCI-S, CPCI-SSH, ESCI Timespan=All years*
# 6	421,158	**ALL FIELDS:** (Psychosis) *OR* **ALL FIELDS:** (Psychotic) *OR* **ALL FIELDS:** (Psychotic disorder*) *OR* **ALL FIELDS:** (Paranoia) *OR* **ALL FIELDS:** (Paranoid) *OR* **ALL FIELDS:** (Delusi*) *OR* **ALL FIELDS:** (Halluci*) *OR* **ALL FIELDS:** (Visual halluci*) *OR* **ALL FIELDS:** (Auditory halluci*) *OR* **ALL FIELDS:** (Multimodal halluci*) *OR* **ALL FIELDS:** (Visual illusion*) *OR* **ALL FIELDS:** (Illusion*) *OR* **ALL FIELDS:** (Imagery) *OR* **ALL FIELDS:** (Schizophrenia spectrum disorder*) *OR* **ALL FIELDS:** (Psychosis spectrum disorder)<br><br>*Indexes=SCI-EXPANDED, SSCI, A&HCI, CPCI-S, CPCI-SSH, ESCI Timespan=All years*
# 5	257,745	**ALL FIELDS:** (Parkinson disease) *OR* **ALL FIELDS:** (Parkinson*) *OR* **ALL FIELDS:** (Parkinsonian) *OR* **ALL FIELDS:** (Parkinsonism) *OR* **ALL FIELDS:** (Atypical parkinsonism)<br><br>*Indexes=SCI-EXPANDED, SSCI, A&HCI, CPCI-S, CPCI-SSH, ESCI Timespan=All years*
# 4	2,833	**ALL FIELDS:** (Parkinson's disease psychosis) *OR* **ALL FIELDS:** (Parkinson disease psychosis)<br><br>*Indexes=SCI-EXPANDED, SSCI, A&HCI, CPCI-S, CPCI-SSH, ESCI Timespan=All years*
# 3	521,870	**ALL FIELDS:** (Voxel-base*) *OR* **ALL FIELDS:** (Voxel-base morphometry) *OR* **ALL FIELDS:** (VBM) *OR* **ALL FIELDS:** (Magnetic resonance spectroscopy) *OR* **ALL FIELDS:** (MRS) *OR* **ALL FIELDS:** (Neuroimaging)<br><br>*Indexes=SCI-EXPANDED, SSCI, A&HCI, CPCI-S, CPCI-SSH, ESCI Timespan=All years*

# 2	713,485	**ALL FIELDS:** (Structual magnetic resonance imaging) *OR* **ALL FIELDS:** (fMRI) *OR* **ALL FIELDS:** (Functional MRI) *OR* **ALL FIELDS:** (Functional magnetic resonance imaging) *OR* **ALL FIELDS:** (Resting state fMRI) *OR* **ALL FIELDS:** (rsfMRI) *OR* **ALL FIELDS:** (Tractography) *OR* **ALL FIELDS:** (DTI) *OR* **ALL FIELDS:** (Diffusion tensor imaging) *OR* **ALL FIELDS:** (Positron emission tomography) *OR* **ALL FIELDS:** (PET) *OR* **ALL FIELDS:** (SPECT) *OR* **ALL FIELDS:** (Single photon emission computed tomography) *OR* **ALL FIELDS:** (Arterial spin labelling)<br><br>*Indexes=SCI-EXPANDED, SSCI, A&HCI, CPCI-S, CPCI-SSH, ESCI Timespan=All years*
# 1	3,503,331	**ALL FIELDS:** (Brain*) *OR* **ALL FIELDS:** (Brain region*) *OR* **ALL FIELDS:** (Functional connect*) *OR* **ALL FIELDS:** (Functional imaging) *OR* **ALL FIELDS:** (Neurophysiological mechanism*) *OR* **ALL FIELDS:** (Neuroanatomical correlate*) *OR* **ALL FIELDS:** (Neural substrate*) *OR* **ALL FIELDS:** (Neural correlate*) *OR* **ALL FIELDS:** (Cerebral mechanism*) *OR* **ALL FIELDS:** (Cerebral atrophy) *OR* **ALL FIELDS:** (Whole brain analys*) *OR* **ALL FIELDS:** (MRI) *OR* **ALL FIELDS:** (Magnetic resonance imaging) *OR* **ALL FIELDS:** (Structural MRI)<br><br>*Indexes=SCI-EXPANDED, SSCI, A&HCI, CPCI-S, CPCI-SSH, ESCI Timespan=All years*

Supplementary Table 1

Study description (including location and scanner details) with statistics (mean, unless otherwise specified) presented for each patient group (PDP patients; PDnP patients). Number of patients for each group are presented alongside gender (males, M; females, F), years of education, scores on cognitive outcomes. Clinical variables of interest are also reported, e.g., motor symptoms and stage (according to the MDS-UPDRS and Hoehn and Yahr stages), PD medications (expressed in mean daily mg where available), and depression scores where available, and global quality assessments for each study.

**Study**	**PDP patient**	**PDnP patients**	**Definition of PD psychosis**	**Age (years)**	**Educations (years)**	**Cognitive outcome**	**PD onset (years)**	**PD duration**	**Hoehn & Yahr stage**	**MDS-UPDRS part III scores (mean)**	**PD medications (mg/day)**	**Depression scores**	**Global quality rating**
						**Diffusion Tensor Imaging (DTI)**							
**Lee et al.** [1] **,** South Korea Philips, 3.0 T	10 (7M, 3F)	14 (5M, 9F)	Semi-structured interview, VH present for > 6 months at time of enrolment	69.2; 66.1	NR	MMSE 27.7; 28.4	NR	7.2; 7.3 (years)	2.2; 2.1	22.9; 20.3	LEDD 981.2 mg; 843.4 mg	NR	7 stars
⸸**Yao et al.** [2]**,** Hong Kong Philips, 3.0 T	12 (10M, 2F)	15 (10M, 5F)	Repetitive complex VH lasting for at least 4 weeks, and occurring at least once every 4 weeks	69.38; 66.34	7.2; 6.3	MMSE 27.625; 29	NR	9.1; 7.1 (years)	3.1; 2.9	19.69; 20	Levodopa 986.9 mg; 689.7 mg	MADRS-S 1.42; 0.63	6 stars
**Zarkali et al.** [3]**,** UK Siemens, 3.0 T	19 (6M, 13F)	81 (47M, 34F)	Presence of VH based on item 1.2 of the UPDRS part I	64.6; 64.4	17.1; 16.9	MMSE 28.6; 28.9	NR	4.8; 4 (years)	NR	29.2; 21.8	LEDD 434.9 mg; 456.9 mg	HADS-D 4.8; 3.8	7 stars
**Hepp et al.** [4]**,** The Netherlands General Electric, 3.0 T	15 (11M, 4F)	40 (21M, 19F)	SCOPA-PC scores of ≥ 1 or 0 on the first item of SCOPA-PC	69; 67	NR	MMSE 26; 28	NR	12; 11 (years)	n=6 stage 2, n=4, stage 2.5, n=5, stage 3; n=16 stage 2, n=6 stage 2.5, n=8 stage 3	37; 30	LEDD 1081 mg; 1008 mg	NR	7 stars
**Lee et al.** [5]**,** South Korea Philips, 3.0 T	10 (7M, 3F)	21 (9M, 11F)	VH definition was based on consensus, with	69.4; 66.2	NR	MMSE 27.6; 28.2	62.2; 59.3	7.2; 7 (years)	2.2; 1.8	22.5; 16.4	LEDD 1031.2 mg; 805.2 mg	GDS 16.6; 15.5	6 stars

			VH persistent for at least 3 months										
⸸**Firbank et al.** [6]**,** UK Philips, 3.0 T	17 (13M, 4F)	19 (17M, 2F)	NPI (hallucination item)	75.5; 72.3	11.6; 11.1	MMSE 23.1; 25.6	NR	11; 9.6 (years)	NR	55.9; 34.7	Levodopa in 24h 717.3 mg; 673. 5 mg	NR	6 stars
**Yuki et al.**[7]**,** Japan Philips, 3.0 T	17 (6M, 11F)	43 (17M, 26F)	Presence of VH (i.e., complex VH that persons, animals and objects were perceived in the absence of an external stimulus), after onset of motor symptoms	73.92; 76.58	NR	MMSE 24.0; 27.28	NR	10.39; 6.42	3.0; 3.0	NR	LEDD 386.63;300	NR	7 stars
**Lenka et al.**[8]**,** India Philips, 3.0 T	42 (35M, 7F)	48 (41M, 7F)	Semi-structured interviews (based on NINDS-NIMH) to explore presence of psychosis	58.5; 57.9	11.3; 12.1	MoCA 25.7; 26.0	51.9; 51.0 (age)	6.5; 5.7 (years)	2.4; 2.3	36.3; 35.2	LEDD 722.8; 577.5	HAM-D 8.6; 6.9	6 stars
**Task-based functional MRI (fMRI)**													
⸸**Firbank et al.** [6]**, UK** Philips, 3.0 T Visual task: checkboard paradigm	17 (13M, 4F)	19 (17M, 2F)	NPI (hallucination item)	75.5; 72.3	11.6; 11.1	MMSE 23.1; 25.6	NR	11; 9.6 (years)	NR	55.9; 34.7	Levodopa in 24h 717.3 mg; 673. 5 mg	NR	6 stars
**Knolle et al.** [9]**,** Germany Siemens, 3.0 T Visual task: odd ball for salience processing	14 (7M, 7F)	23 (14M, 9F)	Presence of psychotic symptoms assessed with the CAARMS and the PANSS	62.5; 63.1	NR	MMSE 28; 29.4	NR	7.7; 9.9 (years)	53.3% stage 1, 20% stage 2, 26.7% stage 3; 61.5% stage 1, 26.9% stage 2, 7.7% stage 3, 0% stage 4, 3.8% stage 5	NR	Levodopa therapy (% yes) 80.8%; 86.7%	BDI 13; 8.1	6 stars

**Lefebvre et al. [10],** France Philips, 3.0 T Visual task: [11] visual perception task	18 (11M, 7F)	16 (12M, 4F)	Presence of VH assessed with the NPI-C	63.5; 62.69	12.44; 13.38	MMSE 28; 28.88	NR	9.06; 8 (years)	2; 2	25; 21.81	LEDD 859.72 mg; 804.25 mg	Hamilton depression scale 3.56; 3.06	7 stars
**Stebbins et al. [12],** US General Electric, 1.5 T Visual task: stroboscopic vs. no visual stimulus, kinematic vs. stationary visual	12 (NR)	12 (NR)	Self-report on presence and frequency of VH. VH present at least three times per week. Severity was assessed with the NPI-Q and SAPS	71.08; 73.25	NR	MMSE 26.17; 27.96	NR	13.92; 11.17 (years)	3; 3	30.42; 31.8	NR	NR	7 stars
**Meppelink et al.[13] ,** The Netherlands Philips, 3.0 T Visual task: detection task	9 (5M, 4F)	14 (11M, 3F)	Presence of complex VH at least weekly during the last month. Severity of VH assessed with the NPI	61.2; 64.6	5.7; 5.7	MMSE 26.8; 27.4	NR	8.1; 8.7 (years)	NR	21.4; 20.4	LEDD 855 mg; 794 mg	NR	6 stars
**Ramirez-Ruiz et al. [14], Spain** General Electric, 1.5 T visual task: one-back repetition detection task as shown by authors	10 (4M, 6F)	10 (4M, 6F)	Presence of VH with a frequency of at least seven times per week	73;72.5	7.8; 6.5	MMSE 25.8; 29.4	NR	11.1; 11 (years)	3.1; 2.5	28.8; 26.8	LEDD 637.5; 585	Hamilton depression scale 7.8; 3.5	6 stars
						**Positron emission tomography (PET)**							
**†Nishio et al. [15],** Japan NR	19 (11M, 8F)	53 (22M, 31F)	NPI	69.4; 65.7	12.7; 11.2	MMSE 26.6; 28.4	NR	7.3; 6.7 (years)	NR	27.2; 16.2	LEDD 612.4 mg; 436.4 mg	NR	5 stars

**†Nishio et al.** [15], Japan NR	17 (7M, 10F)	53 (22M, 31F)	NPI	67.3; 65.7	13.4; 11.2	MMSE 27.4; 28.4	NR	6.5; 6.7 (years)	NR	18,7; 16.2	LEDD 516.3; 612.4 mg	NR	5 stars
**†Nishio et al.** [15], Japan NR	24 (12M, 12F)	53 (22M, 31F)	NPI	68.5; 65.7	13; 11.2	MMSE 27.4; 28.4	NR	8.5; 6.7 (years)	NR	23.1; 16.2	LEDD 53 mg; 612.4 mg	NR	5 stars
**Park et al.** [16], South Korea Siemens, NR	7 (3M, 4F)	13 (8M, 5F)	VH assessed using the NPI administered by a neurologist	71; 66.3	9.1; 9.2	MMSE 26.1; 26.9	NR	5.4; 5.1 (years)	2; 1.5	26.8; 22.1	LEDD 537.1 mg; 531.4 mg	GDS 15.4; 13.3	6 stars
**Boecker et al.** [17], Germany ECAT, EXACT HR +, CTI PET system	8 (5M, 3F)	11 (8M, 3F)	NPI (hallucination item)	72.88; 70.56	NR	MMSE 25.75; 26.82	NR	11; 8.05 (years)	3.31; 2.68	46.25; 32.73	Levodopa equivalent dose 667 mg; 617 mg	NR	5 stars
						**Resting state functional MRI (rsfMRI)**							
**‡Yao et al.** [2], Hong Kong Philips, 3.0 T	12 (10M, 2F)	15 (10M, 5F)	Repetitive complex VH lasting for at least 4 weeks, and occurring at least once every 4 weeks	69.38; 66.34	7.2; 6.3	MMSE 27.625; 29	NR	9.1; 7.1 (years)	3.1; 2.9	19.69; 20	Levodopa 986.9 mg; 689.7 mg	MADRS-S 1.42; 0.63	6 stars
**Shine et al.** [18], Australia General Electric, 3.0 T	10 (NR)	9 (NR)	Scores on the BPP	69.5; 67.1	NR	MoCA 26; 27.6	NR	6.9; 4.4 (years)	NR	34; 32	DDE (dopamine dose equivalent) 819.5 mg; 512.5 mg	BDI-II 15.5; 8.9	5 stars
**Yao et al.** [19], Hong Kong Philips, 3.0 T	12 (3M, 9F)	12 (4M, 8F)	VH assessed with PPRS	67.6; 63.4	NR	MMSE 27.6; 28.5	NR	10; 8.4 (years)	3.2; 2.8	20.9; 18	LEDD 978.7 mg; 704.9 mg	NR	6 stars
**Bejr-Kasem et al.** [20], Spain Philips, 3.0 T	18 (10M, 8F)	14 (10M, 4F)	PD patients included if VH remained stable during the 3 months before inclusion in the study	70.4; 65.8	12.5; 11.6	PD-CRS, 91.9; 92.9	NR	5.2; 4 (years)	2.1; 2.1	21.9; 25.8	LEDD 697.2 mg; 601.1 mg	HADS-D 2.2; 3.3	7 stars

† **Marques et al.** [21] Group: Visual illusions France General Electric, 3.0 T	19 (10M, 9F)	23 (14M, 9F)	VH and VI were defined using the SCPOA-PC, VI or VH had to occur at least once a week within the past 3 months	68.31; 69.21	17.9; 17.72	MoCA, 26.4; 25.88	NR	9; 6.21 (years)	2.5; 2.32	27.89; 28.69	LEDD 1061.36 mg; 953.49 mg	NR	5 stars
† **Marques et al.** [21] Group: VH France General Electric, 3.0 T	20 (12M, 8F)	23 (14M, 9F)	VH and VI were defined using the SCPOA-PC, VI or VH had to occur at least once a week within the past 3 months	70.2; 69.21	18.95; 17.72	MoCA, 25.53; 25.88	NR	11.15; 6.21 (years)	2.9; 2.32	33.9; 28.69	LEDD 1012.79 mg; 953.49 mg	NR	5 stars
**Diez-Cirarda et al.** [22] Spain Philips, 3.0 T	12 (5M, 7F)	35 (22M, 13F)	PD with VH were classified according to the NEVHI	59.0; 63.5	10.3; 9.5	MoCA, 23.2; 23.6	NR	8.4; 6.4	2.5; 2.5	33.0; 26.9	LEDD 795.4 mg; 613.4 mg	GDS 4.7; 3.2	5 stars
**Thomas et al.** [23] UK Siemens, 3.0 T	15 (4M, 11F)	75 (44M, 31F)	VH were classified using question 1.2 from the MDS-UPDRS part I when patients scored >1	65.33; 64.12	17.77; 16.83	MoCA, 27.60; 28.15	NR	4.67; 3.83 (years)	NR	25.40; 21.89	LEDD 421.67 mg; 431.89 mg	HADS depression, 4.73; 3.97	7 stars
**Zhong et al.** [24] China Siemens, 3.0 T	23 (6M, 17F)	35 (13M, 22F)	VH were classified using question 1.2 from the MDS-UPDRS part I when patients scored >1 and had VH were stable in the last months since screening	60.57; 61.06	10.35; 8.63	MoCA, 26.0; 25.46	NR	7.08; 5.06 (years)	2.59; 2.31	32.78; 30.97	LEDD 655.46 mg; 459.35 mg	HAM-D 9.70; 8.77	7 stars

**Zarkali et al.**[25], UK, rsfMRI (Siemens, 3.0 T)	16 (5M, 11F)	75 (41M, 34F)	VH were classified based if patients scored ≥1 MDS-UPDRS part 1 question 1.2.	64.8; 64.4	17.5; 16.9	MMSE 28.6; 28.9 MoCA 26.9; 28.2	NR	5.3; 3.9 (years)	NR	29.8; 21.2	LEDD 450.0; 437.0	HADS depression, 4.7; 3.9	7 stars
**Miloserdov et al.**[26]**,** Germany, rsfMRI (Siemens, 3.0 T)	16 (11M, 5F)	16 (12M, 4F)	Severity of hallucinations was measured with the UM-PDHQ	70.50; 70.19	3.53; 3.59 (years of secondary education)	MMSE 28.31; 28.87	NR	9.78; 4.61 (years)	2.38; 1.88	26.63; 20.94	LEDD 797.98; 385.53	BDI 9.13; 7.75	6 stars
**Hepp et al.**[27]**,** The Netherlands, rsfMRI (General Electric, 3.0 T)	15 (11M, 4F)	40 (21M, 19F)	Presence of VH was assessed using the SCOPA-PC	69; 67	NR	MMSE 26; 28	NR	12; 11 (years)	stage 2, n=6, stage 2.5, n=4, stage 3, n=5; stage 2, n=16, stage 2.5, n=16, stage 3, n=8	37; 30	LEDD 1081; 1008	NR	7 stars
**Walpola et al.**[28]**,** Australia, rsfMRI (General Electric, 3.0 T)	18 (14M, 4F)	20 (17M, 3F)	Presence of VH was assessed using the MDS-UPDRS part 1 question 1.2, if patients scored ≥1 on question 1.2	67.5; 63.7	13.3; 14.6	MoCA 27.9; 27.9	NR	7.6; 5.7 (years)	2.2; 2.1	33.4; 28.4	DDE 832.8; 691.5	BDI-II 11.6; 8.1	7 stars
**Single photon emission computed tomography (SPECT)**													
**Kiferle et al.** [29]**,** Italy General Electric, NR	18 (NR)	18 (NR)	VH established with the NINDS-NIMH criteria	75; 76.6	NR	MMSE 24.9; 25.4	69.5; 70.8	66; 69.3 (months)	NR	27.3; 25.8	LEDD 547.3 mg; 496.2 mg	NR	5 stars
**Jaakkola et al.** [30]**,** Finland General Electric, NR	22 (13M, 9F)	48 (27M, 21F)	VH developed after baseline at follow up	66; 64	NR	NR	NR	7; 6.7 (years)	3.6; 3	NR	LEDD 664 mg; 683 mg	NR	8 stars
**Magnetic resonance spectroscopy (MRS)**													
**⸸Firbank et al.** [6]**, UK**	17 (13M, 4F)	19 (17M, 2F)	NPI (hallucination item)	75.5; 72.3	11.6; 11.1	MMSE 23.1; 25.6	NR	11; 9.6 (years)	NR	55.9; 34.7	Levodopa in 24h 717.3 mg; 673. 5 mg	NR	6 stars

Philips, 3.0 T<br>Visual task:<br>checkboard<br>paradigm

† Studies that included one or more groups of PD psychosis patients with different symptoms, e.g., visual illusions, visual hallucinations.
‡ Studies with more than one neuroimaging modality.

BDI: Beck Depression Inventory; BPP: Bistable Percept Paradigm; CAARMS: Comprehensive Assessment of At Risk Mental States; GDS: Geriatric Depression Scale; HADS-D: Hospital Anxiety and Depression Scale – depression item; HADS-A: Hospital Anxiety and Depression Scale – anxiety item; HAMD-D: Hamilton Depression Scale; LEDD: Levodopa equivalent daily dose; MADRS: Montgomery-Asberg Depression Rating Scale; MDS-UPDRS: Movement Disorders Society Unified Parkinson's Disease Rating Scale; MMSE: Mini-Mental State Examination; MoCA: Montreal Cognitive Assessment; NEVHI: North-East Visual Hallucinations Interview; NPI: Neuropsychiatric Inventory; NPI-C: Neuropsychiatric Inventory – Clinician; NPI-Q: Neuropsychiatric Inventory – Questionnaire; NR: None reported; PANSS: Positive and Negative Symptom Scale; PD-CRS: Parkinson's Disease – Cognitive Rating Scale; PPRS: Parkinson Psychosis Rating Scale; VH: visual hallucinations.

Supplementary Table 2

Quality rating of the 29 studies included in the review. Study quality was assessed with the Newcastle-Ottawa Scale in the three methodological domains, i.e., Selection, Comparability, and Exposure. Studies were assigned a maximum of one star per item with the exception of Comparability (i.e., maximum of two stars).

**Study**	**Selection**				**Comparability**	**Exposure**		
	**Is the case definition adequate?**	**Representativeness of the cases**	**Selection of controls**	**Definition of Controls**	**Comparability of cases and controls on the basis of the design and analysis**	**Ascertainment of exposure**	**Same method of ascertainment for cases and controls**	**Non-response rate**
**Kiferle et al.[29]**	*		*		*	*	*	
**Lee et al. [1]**	*		*	*	**	*	*	
**Yao et al. [2]**	*		*		**	*	*	
**Shine et al. [18]**	*		*		*	*	*	
**Nishio et al. [15]**	*		*		*	*	*	
**Park et al. [16]**	*		*		**	*	*	
**Stebbins et al. [12]**	*		*	*	**	*	*	
**Yao et al. [19]**	*		*		**	*	*	
**Zarkali et al. [3]**	*		*	*	**	*	*	
**Hepp et al. [4]**	*		*	*	**	*	*	
**Bejr-Kasem et al. [20]**	*		*		**	*	*	*
**Lee et al. [5]**	*		*		**	*	*	
**Lefebvre et al. [10]**	*		*	*	**	*	*	
**Boecker et al. [17]**	*		*		*	*	*	
**Knolle et al. [9]**	*		*		**	*	*	
**Jakkola et al. [30]**	*	*	*		**	*	*	*
**Firbank et al. [6]**	*		*		**	*	*	

**Meppelink et al. [13]**	*	*		**	*	*	
**Marques et al. [21]**	*	*		*	*	*	
**Thomas et al. [23]**	*	*	*	**	*	*	
**Zhong et al. [24]**	*	*		**	*	*	*
**Diez-Cirarda et al. [22]**	*			**	*	*	
**Hepp et al.[27]**	*	*	*	**	*	*	
**Ramirez-Ruiz et al. [14]**	*	*		**	*	*	
**Zarkali et al. [25]**	*	*	*	**	*	*	
**Miloserdov et al. [26]**	*	*		**	*	*	
**Walpola et al. [28]**	*	*	*	**	*	*	
**Lenka et al. [8]**	*	*		**	*	*	
**Yuki et al. [7]**	*	*	*	**	*	*	

References:

1. Lee J-Y, Yoon EJ, Lee WW, Kim YK, Lee J-Y, Jeon B. Lateral geniculate atrophy in Parkinson's with visual hallucination: A trans-synaptic degeneration? *Movement Disorders*. 2016;31(4):547-554. doi:https://doi.org/10.1002/mds.26533
2. Yao NL, Cheung C, Pang S*, et al*. Multimodal MRI of the hippocampus in Parkinson's disease with visual hallucinations. Article. *Brain Struct Funct*. Jan 2016;221(1):287-300. doi:10.1007/s00429-014-0907-5
3. Zarkali A, McColgan P, Ryten M*, et al*. Differences in network controllability and regional gene expression underlie hallucinations in Parkinson's disease. *Brain : a journal of neurology*. 2020;29doi:http://dx.doi.org/10.1093/brain/awaa270
4. Hepp DH, Foncke EMJ, Berendse HW*, et al*. Damaged fiber tracts of the nucleus basalis of Meynert in Parkinson's disease patients with visual hallucinations. Article. *Scientific Reports*. Aug 2017;7doi:10.1038/s41598-017-10146-y
5. Lee WW, Yoon EJ, Lee JY, Park SW, Kim YK. Visual Hallucination and Pattern of Brain Degeneration in Parkinsons Disease. Article. *Neurodegener Dis*. 2017;17(2-3):63-72. doi:10.1159/000448517
6. Firbank MJ, Parikh J, Murphy N*, et al*. Reduced occipital GABA in Parkinson disease with visual hallucinations. *Neurology*. 2018;91(7):e675. doi:10.1212/WNL.0000000000006007
7. Yuki N, Yoshioka A, Mizuhara R, Kimura T. Visual hallucinations and inferior longitudinal fasciculus in Parkinson's disease. *Brain and Behavior*. 2020;10(12):e01883.
8. Lenka A, Ingalhalikar M, Shah A*, et al*. Abnormalities in the white matter tracts in patients with Parkinson disease and psychosis. *Neurology*. May 5 2020;94(18):e1876-e1884. doi:10.1212/wnl.0000000000009363
9. Knolle F, Garofalo S, Viviani R*, et al*. Altered subcortical emotional salience processing differentiates Parkinson's patients with and without psychotic symptoms. *Neuroimage Clin*. 2020;27:102277. doi:10.1016/j.nicl.2020.102277
10. Lefebvre S, Baille G, Jardri R*, et al*. Hallucinations and conscious access to visual inputs in Parkinson's disease. *Scientific Reports*. 2016/11/14 2016;6(1):36284. doi:10.1038/srep36284
11. Pins D, ffytche D. The Neural Correlates of Conscious Vision. *Cerebral Cortex*. 2003;13(5):461-474. doi:10.1093/cercor/13.5.461
12. Stebbins GT, Goetz CG, Carrillo MC*, et al*. Altered cortical visual processing in PD with hallucinations - An fMRI study. Article. *Neurology*. Oct 2004;63(8):1409-1416.
13. Meppelink AM, De Jong BM, Renken R, Leenders KL, Cornelissen FW, Van Laar T. Impaired visual processing preceding image recognition in Parkinson's disease patients with visual hallucinations. *Brain*. 2009;132(11):2980-2993.
14. Ramírez-Ruiz B, Martí MJ, Tolosa E*, et al*. Brain response to complex visual stimuli in Parkinson's patients with hallucinations: a functional magnetic resonance imaging study. *Movement disorders: official journal of the Movement Disorder Society*. 2008;23(16):2335-2343.
15. Nishio Y, Yokoi K, Hirayama K*, et al*. Defining visual illusions in Parkinson's disease: Kinetopsia and object misidentification illusions. Article. *Parkinsonism & Related Disorders*. Oct 2018;55:111-116. doi:10.1016/j.parkreldis.2018.05.023
16. Park HK, Kim JS, Im KC*, et al*. Visual hallucinations and cognitive impairment in parkinson's disease. *Canadian Journal of Neurological Sciences*. 01 Sep 2013;40(5):657-662. doi:http://dx.doi.org/10.1017/S0317167100014888
17. Boecker H, Ceballos-Baumann AO, Volk D, Conrad B, Forstl H, Haussermann P. Metabolic alterations in patients with Parkinson disease and visual hallucinations. *Archives of Neurology*. 2007;64(7):984-988.
18. Shine JM, Keogh R, O'Callaghan C, Muller AJ, Lewis SJG, Pearson J. Imagine that: elevated sensory strength of mental imagery in individuals with Parkinson's disease and visual hallucinations. Article. *Proc R Soc B-Biol Sci*. Jan 2015;282(1798)doi:10.1098/rspb.2014.2047

19. Yao NL, Chang RSK, Cheung C, *et al*. The Default Mode Network is Disrupted in Parkinson's Disease with Visual Hallucinations. Article. *Human Brain Mapping*. Nov 2014;35(11):5658-5666. doi:10.1002/hbm.22577
20. Bejr-Kasem H, Pagonabarraga J, Martinez-Horta S, *et al*. Disruption of the default mode network and its intrinsic functional connectivity underlies minor hallucinations in Parkinson's disease. Article. *Movement Disorders*. Jan 2019;34(1):78-86. doi:10.1002/mds.27557
21. Marques A, Taylor NL, Roquet D, *et al*. Structural and functional correlates of hallucinations and illusions in Parkinson’s Disease. *Journal of Parkinson's disease*. 2022;12(1):397-409.
22. Diez-Cirarda M, Cabrera-Zubizarreta A, Murueta-Goyena A, *et al*. Multimodal visual system analysis as a biomarker of visual hallucinations in Parkinson’s disease. *J Neurol*. 2022:1-11.
23. Thomas GE, Zeidman P, Sultana T, Zarkali A, Razi A, Weil RS. Changes in both top-down and bottom-up effective connectivity drive visual hallucinations in Parkinson’s disease. *Brain Commun*. 2023;5(1):fcac329.
24. Zhong M, Li C, Lu H, *et al*. Aberrant gray matter volume and functional connectivity in Parkinson’s disease with minor hallucination. *Frontiers in Aging Neuroscience*. 2022;14
25. Zarkali A, Luppi AI, Stamatakis EA, *et al*. Changes in dynamic transitions between integrated and segregated states underlie visual hallucinations in Parkinson’s disease. *Communications Biology*. 2022;5(1):928.
26. Miloserdov K, Schmidt-Samoa C, Williams K, *et al*. Aberrant functional connectivity of resting state networks related to misperceptions and intra-individual variability in Parkinson‘s disease. *NeuroImage: Clinical*. 2020;25:102076.
27. Hepp DH, Foncke EM, Olde Dubbelink KT, van de Berg WD, Berendse HW, Schoonheim MM. Loss of functional connectivity in patients with Parkinson disease and visual hallucinations. *Radiology*. 2017;285(3):896-903.
28. Walpola IC, Muller AJ, Hall JM, *et al*. Mind-wandering in Parkinson's disease hallucinations reflects primary visual and default network coupling. *Cortex*. 2020;125:233-245.
29. Kiferle L, Ceravolo R, Giuntini M, *et al*. Caudate dopaminergic denervation and visual hallucinations: evidence from a ^{123}I-FP-CIT SPECT study. *Parkinsonism Relat Disord*. Jul 2014;20(7):761-5. doi:10.1016/j.parkreldis.2014.04.006
30. Jaakkola E, Joutsa J, Makinen E, Johansson J, Kaasinen V. Ventral striatal dopaminergic defect is associated with hallucinations in Parkinson's disease. *Eur J Neurol*. Nov 2017;24(11):1341-1347. doi:10.1111/ene.13390
